# Closure of the neuro‐central synchondrosis and other physes in foal cervical spines

**DOI:** 10.1111/evj.14093

**Published:** 2024-04-09

**Authors:** Kristin Olstad, Mari Dahl Bugge, Bjørnar Ytrehus, Anne Selvén Kallerud

**Affiliations:** ^1^ Department of Companion Animal Clinical Sciences, Equine Section Faculty of Veterinary Medicine, Norwegian University of Life Sciences Ås Norway; ^2^ Department of Biomedical Science and Veterinary Public Health, Pathology Unit Swedish University of Agricultural Sciences Uppsala Sweden

**Keywords:** ataxia, computed tomography, horse, osteochondrosis, stenosis, vertebral growth

## Abstract

**Background:**

The neuro‐central synchondrosis (NCS) is a physis responsible for the growth of the dorsal third of the vertebral body and neural arches. When the NCS of pigs is tethered to model scoliosis, stenosis also ensues. It is necessary to describe the NCS for future evaluation of its potential role in equine spinal cord compression and ataxia (wobbler syndrome).

**Objectives:**

To describe the NCS, including when it and other physes closed in computed tomographic (CT) scans of the cervical spine of foals, due to its potential role in vertebral stenosis.

**Study design:**

Post‐mortem cohort study.

**Methods:**

The cervical spine of 35 cases, comprising both sexes and miscellaneous breeds from 153 gestational days to 438 days old, was examined with CT and physes scored from 6: *fully open* to 0: *fully closed*. The dorsal physis, physis of the dens and mid‐NCS were scored separately, whereas the cranial and caudal NCS portions were scored together with the respective cranial and caudal vertebral body physes.

**Results:**

The NCS was a pair of thin physes located in a predominantly dorsal plane between the vertebral body and neural arches. The mid‐NCS was closed in C1 from 115 days of age, and in C2–C7 from 38 days of age. The dorsal physis closed later than the NCS in C1, and earlier than the NCS in C2–C7. The dens physis was closed from 227 days of age. The cranial and caudal physes were closing, but not closed from different ages in the different vertebrae of the oldest cases.

**Main limitations:**

Hospital population.

**Conclusions:**

The NCS was a thin physis that contributed mainly to height‐wise growth, but also width‐ and length‐wise growth of the vertebral body and neural arches. The mid‐NCS was closed in all cervical vertebrae from 115 days of age. The NCS warrants further investigation in the pathogenesis of vertebral stenosis.

## Resumen

**Historial:**

La sincondrosis neuro‐central (NCS) es la fisis responsable del crecimiento del tercio dorsal del cuerpo vertebral y de los arcos neurales. Cuando la NCS en cerdos se asocia a un modelo de escoliosis, también se produce estenosis. Es necesario describir la NCS para la futura evaluación de su rol potencial en la comprensión de la medula espinal equina y ataxia (síndrome de Wobbler).

**Objetivos:**

Describir la NCS incluyendo cuando ella y otras fisis se cierran, por tomografía computarizada (CT) de la columna cervical de potrillos, debido a su rol potencial en la estenosis vertebral.

**Diseño del Estudio:**

Estudio de cohorte post‐mortem.

**Métodos:**

La columna cervical de 35 casos, incluyendo ambos sexos y diferentes razas, desde 153 días gestacionales hasta 438 días de edad, fueron examinadas por CT y las fisis fueron dadas un puntaje de, 6: completamente abiertas a, 0: completamente cerradas. La fisis dorsal, la fisis del hueso odontoides y NCS media fueron evaluadas en forma separada, mientras las porciones de NCS craneal y caudal fueron evaluadas juntas con las respectiva fisis del cuerpo vertebral craneal y caudal.

**Resultados:**

La NCS es un par de fisis delgadas localizadas predominantemente en el plano dorsal entre el cuerpo vertebral y los arcos vertebrales. La NCS media estaba cerrada en C1 desde los días 115 de edad, y en C2‐C7 a partir de los 38 días de edad. La fisis dorsal se cerró más tarde que la NCS en C1, y antes que la NCS en C2‐C7. La fisis del hueso odontoides estaba cerradas a partir de los 227 días de edad. Las fisis craneal y caudal estaban cerrándose, pero no estaban cerradas a distintas edades en las diferentes vertebras en los casos mayores de edad.

**Limitaciones principales:**

Población de hospital

**Conclusiones:**

La NCS es una fisis delgada que contribuye principalmente al crecimiento en altura, pero también en ancho y largo del cuerpo vertebral y arcos vertebrales. La NCS media estaba cerrada en todas las vértebras cervicales a partir de los 115 días de edad. La NCS merece ser investigada más en la patogénesis de la estenosis vertebral.

**Palabras Clave:** ataxia, tomografía computarizada, caballo, osteocondrosis, estenosis, crecimiento vertebral

## 1 INTRODUCTION

**FIGURE 1:**
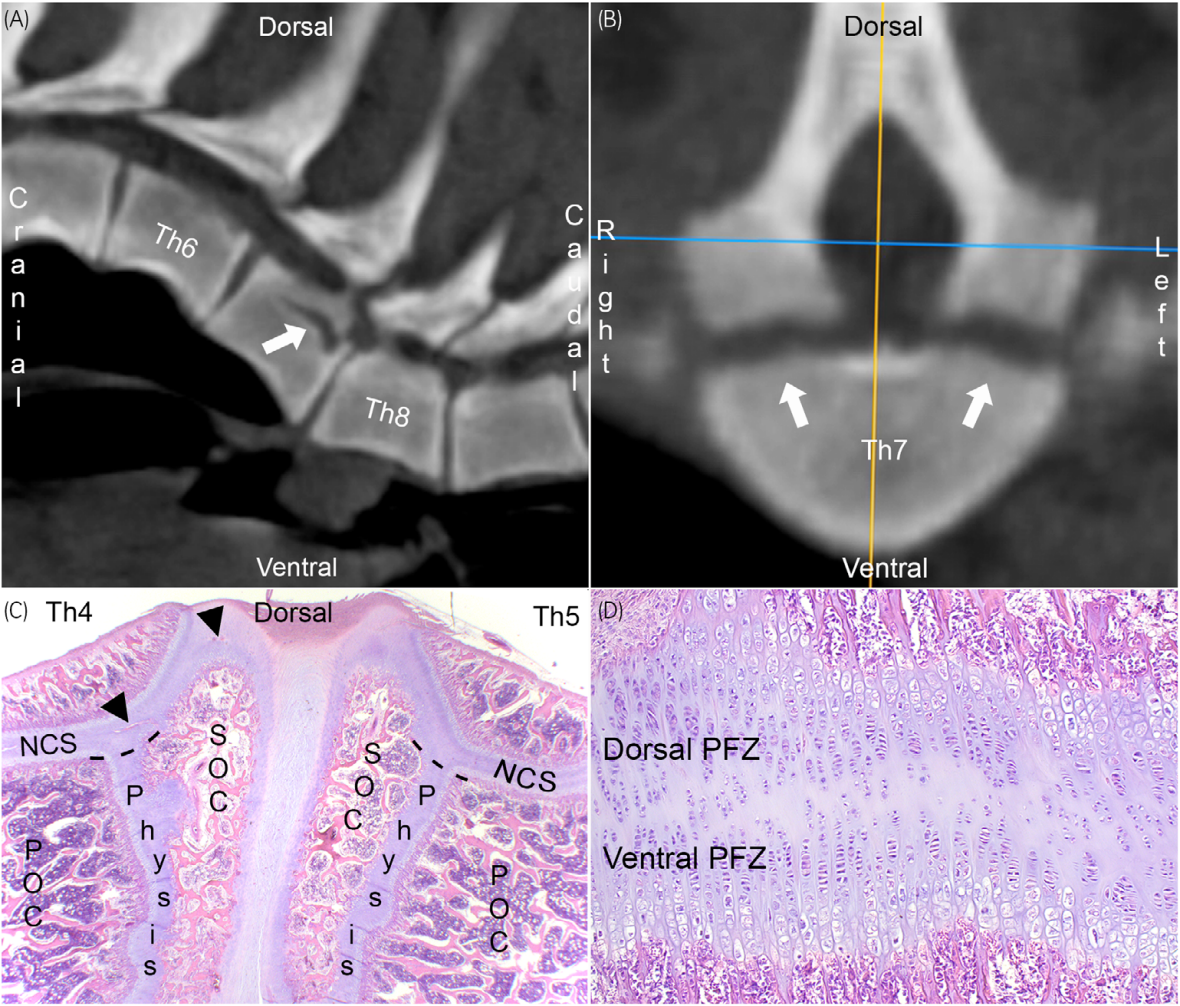
Neuro‐central synchondrosis in pigs. (A) Computed tomographic (CT) scan of a 100 kg Landrace boar, parasagittal slice. The hypoattenuating line in Th7 (arrow) was suspected of representing fracture secondary to adjacent vertebral wedging. (B) Transverse slice through Th7 from (A). The hypoattenuating line is symmetric (arrows) and literature search revealed that it runs through a pair of physes between the vertebral body and each neural arch known as the neuro‐central synchondroses (NCSs). (C) Parasagittal histological section from Th4‐5 of a 12 kg mixed‐breed piglet, haematoxylin and eosin staining. The vertebral bodies have a primary (POC), a cranial and a caudal secondary ossification centre (SOC). The physes between these centres are responsible for growth of the ventral two‐thirds of the vertebral body (below dashed lines), whereas the NCS is responsible for growth of the dorsal third of the vertebral body and the neural arches (above dashed lines). The growth cartilage contains blood vessels (arrowheads). (D) Magnified view from (C). The middle portion of the NCS has a central resting zone and one set of proliferative (PFZ), hypertrophic and mineralised zones in each of the dorsal and ventral directions. Modified and reprinted with permission from Olstad et al.^1^

During a computed tomographic (CT) study of porcine juvenile kyphosis,^1^ a gradually widening hypoattenuating line was detected (Figure 1A) and suspected of representing partial fracture secondary to adjacent vertebral wedging. However, the line was symmetric (Figure 1B) and literature search revealed that it ran through the caudal portion of a physis known as neuro‐central synchondrosis (NCS).^1^ The NCS is a paired structure located between each neural arch and the vertebral body or centrum.^2^, ^3^ Synchondroses are hyaline cartilage joints, and temporary synchondroses like the NCS are physes.^2^, ^3^ Most vertebral bodies have a primary ossification centre, one cranial and one caudal secondary ossification centre (Figure 1C). The physes between these centres (Figure 1C) are responsible for the growth of the ventral two‐thirds of the vertebral body.^2^, ^3^ The NCS (Figure 1C) is responsible for the growth of the dorsal third of the vertebral body and the neural arches.^2^, ^3^ The NCS has a central resting zone and one set of proliferative, hypertrophic and mineralised growth cartilage zones in each of the dorsal and ventral directions (Figure 1D).^1^

The NCS of animals is barely mentioned in veterinary literature,^4^, ^5^ but extensively described in human literature because surgeons use it to model scoliosis in children.^6^ The spine of growing pigs is tethered until the desired scoliosis angle develops and then corrected.^6^, ^7^ One of the tethering techniques involves placing screws across the NCS,^6^, ^7^ resembling transphyseal bridging. Unilateral NCS bridging results in scoliosis, lordosis, axial rotation and shortening of the pedicle on the tethered side, or: stenosis.^7^ Stenosis is one of several mechanisms implicated in spinal cord compression and ataxia of young horses (wobbler syndrome), along with cervical vertebral malformation and instability.^8^ Experimental tethering does not clarify how spontaneous lesions occur, but a case report exists of a single foal with ischaemic necrosis of what is described as ‘the physis that runs parallel to the vertebral canal’ and corresponds to the NCS in the figures.^9^ Failure of the blood supply to limb epiphyseal^10^ and spinal growth cartilages^11^, ^12^ causes ischaemic chondronecrosis, which, in turn, leads to a delay in endochondral ossification or osteochondrosis. Observation of ischaemic chondronecrosis^9^ indicates that vascular failure and osteochondrosis occur in the equine NCS, and experimental tethering confirms that delayed growth of this structure leads to stenosis.^7^ It is therefore necessary to study the blood supply to the NCS (Figure 1C),^10^ and the first step is to determine when growth cartilage is present in vertebrae. The aim of the current exploratory study was to describe the NCS, including when it and other physes closed in CT scans of the cervical spine of foals, due to its potential role in vertebral stenosis.

## 2 MATERIALS AND METHODS

### 2.1 Population

The study population was recruited via the teaching hospitals of the Norwegian University of Life Sciences (NMBU) and the Swedish University of Agricultural Sciences (SLU) during 2021–2023. Foetuses and foals of any breed and sex up to 14 months old were collected (Table S1). Recorded information about gestation length, cause of death, systemic and orthopaedic conditions, including bacterial culture results, was gathered. Cases were numbered 1–35 by increasing age, or by increasing size if age was equal or unrecorded (Table S1). Cases that were premature or dysmature (criterion: gestation length different from 332 days)^13^ according to the owner or clinical records were labelled ‘p’ or ‘d’, respectively (Table S1).

### 2.2 Sample collection

At NMBU, whole bodies were collected from 14 cases and intact necks were collected from 9 cases, whereas at SLU, most soft tissue was removed from the last 12 necks by dissection (Table S1). All samples were frozen with the necks as straight as possible and remained frozen during CT scanning to limit decomposition for future histological validation.

### 2.3 CT scanning

At NMBU, a 20‐slice CT scanner (Siemens Somatom Confidence RT Pro 20) was used, whereas at SLU, a 64‐slice CT scanner (Siemens Somatom Definition AS) was used. The acquisition protocol was identical in both locations: 120 kV, automated mAs up to 450 and slice thickness 0.6 mm, but longer increment (1 vs. 0.3 mm), pitch (0.75 vs. 0.5) and rotation time (1 vs. 0.5 s) were used for whole‐body than neck‐only scans. Combined with the different field of view sizes, a pixel spacing of 0.9 mm was achieved for whole‐body scans, whereas pixel spacing was 0.4 and 0.3 mm for intact and dissected necks, respectively. Scans were reconstructed using soft tissue and bone kernels.

### 2.4 Factors affecting evaluation

Scans were imported into Horos software version 4.0.0 RC5 (www.horosproject.com), and various factors potentially affecting radiological evaluation were assessed, including decomposition, dissection, collimation, positioning, artefacts and asymmetry of the ventral laminae of C6 (e.g., transposition to adjacent vertebrae). Decomposition was defined as randomly distributed, focal hypoattenuating defects in bone marrow or cortex without additional radiographic signs of diseases like osteochondrosis.^14^

### 2.5 Measurement of vertebral length

Using multi‐planar reconstruction, the dorsal plane was aligned with the floor of the spinal canal of the vertebra to be measured, and the sagittal and transverse planes divided the vertebra into equal halves. The maximal craniocaudal length of the mineralised portion of the vertebral body (or ventral arch in C1), including secondary ossification centres, was measured at midway the dorso‐ventral height of the body.

### 2.6 Qualitative evaluation of radiological maturity

The CT scans of the cervical spine were evaluated in three orthogonal planes and as three‐dimensional (3D) volume‐rendered models. Standardised views of 3D models of the vertebrae were printed on paper to facilitate sorting cases in order of radiological maturity.

Ranking of radiological maturity focused on the presence of ossification centres, articular and transverse processes, the ventral laminae of C6 and the dorsal spinous process of C7. When present, features were ranked in terms of size, shape and contours, for example: small ossification centres were ranked as less mature than large centres, generic spherical‐shaped centres were ranked as less mature than centres with a shape resembling the mature bone, and centres with diffuse/soft contours were ranked as less mature than centres with sharp contours. Small mineralised foci with spherical shape were referred to as ossification nuclei, whereas larger mineralised foci with a shape resembling the mature bone were referred to as ossification centres. Bone contours facing articular surfaces or physes, that is, ossification fronts, were evaluated for the presence of millimetre‐sized cylindrical protrusions referred to as tubes and shallow, pinprick‐sized indentations referred to as dimples (fig. 2 in Olstad et al.^14^).

The description is focused on observations interpreted to represent normal development because they were generalised, symmetric and present in several cases across an age range. Changes interpreted to represent lesions because they were focal, variably symmetric and sometimes included additional signs of diseases like osteochondrosis^14^ were excluded from this report.

### 2.7 Scoring of physeal closure

A physis was defined as the hypoattenuating area or line between two ossification centres. Epiphyseal growth cartilage superficial to one ossification centre was not scored.

All included physes were scored because decomposition in bone marrow did not interfere with scoring, and the only changes suspected of representing lesions were focal, in which case the foci were ignored and the rest of the physis was scored.

**FIGURE 2:**
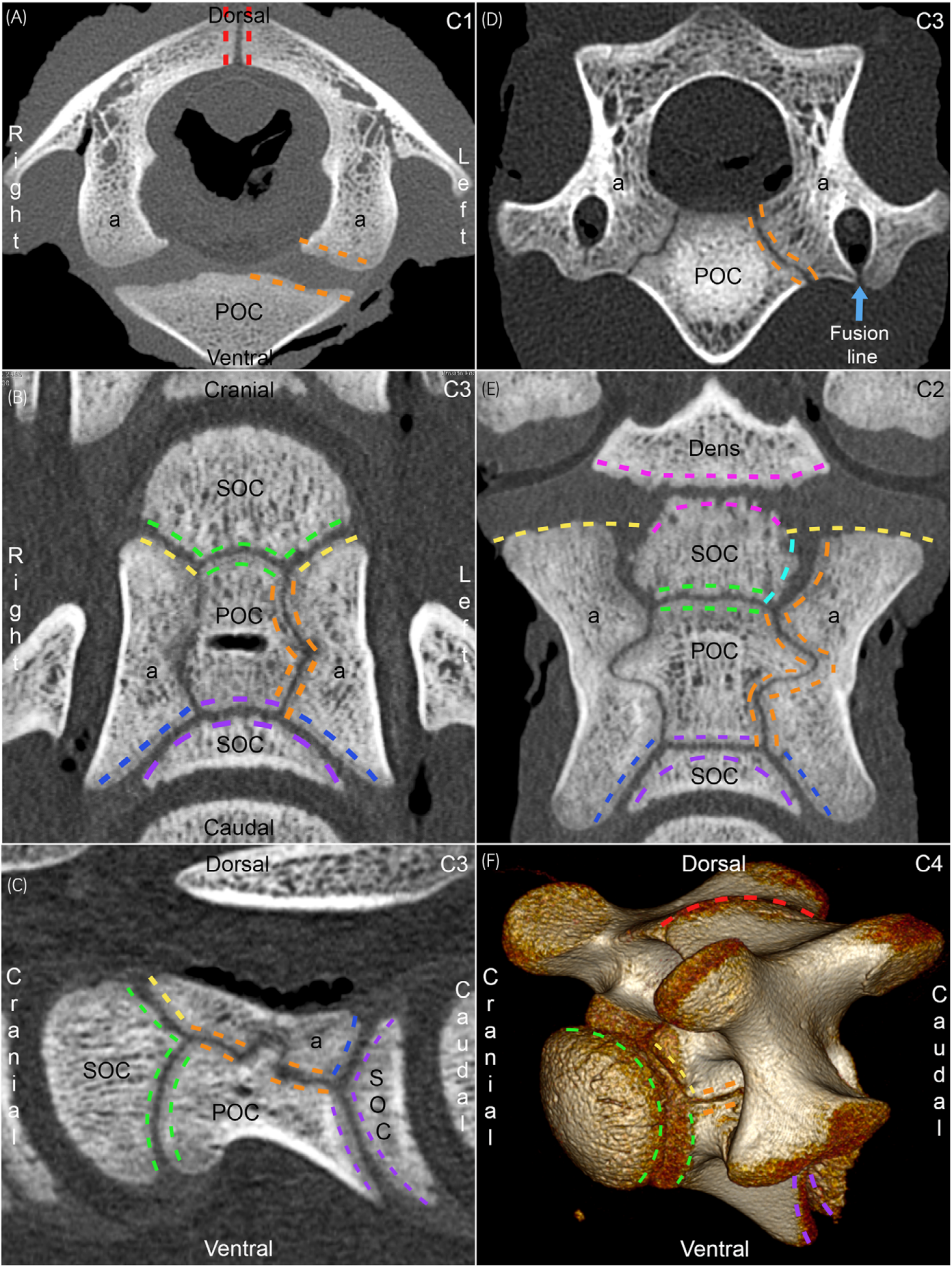
Grouping of physes for scoring. All 2D images are from case 16, 0 days/19 h old, figure (F) is from case 27d, 65 days old. (A) Transverse slice from C1. Three physes were scored in C1: the (mid‐)dorsal physis (red dashed lines), left (orange lines) and right neuro‐central synchondroses. POC, primary ossification centre; a, neural arch. (B) Dorsal slice from C3. In C3 (shown) to C7, five physes were scored: the dorsal physis (not shown), left and right NCSs, cranial and caudal physes of the vertebral body. The mid‐portion of the NCS (orange lines) was scored on its own, whereas the cranial portion of the NCS was grouped with the cranial physis (green and yellow lines), and the caudal portion of the NCS was grouped with the caudal physis (blue and purple lines) for scoring. The mid‐portion of the NCS is craniocaudally oriented, whereas the cranial and caudal portions are axio‐laterally obliquely oriented. SOC, secondary ossification centre. Remainder of legend, see 2A. (C) Sagittal slice from C3, same colour codes as (B). The mid‐portion of the NCS is craniocaudally oriented, whereas the cranial and caudal portions are (axio‐)ventro‐dorsally obliquely oriented. Note that the NCS makes a dorsally‐pointing V‐shaped deviation midway between cranial and caudal. (D) Transverse slice from C3, same orientation as (A) and same colour codes as (B). The mid‐portion of the NCS is axio‐dorsally to ventro‐laterally obliquely oriented. There is a hypoattenuating line (arrow) where the lateral portion fuses with the medial portion of the primary ossification centre ventral to the transverse foramen. (E) Dorsal slice from C2, same orientation as (B). Six physes were scored in C2: the dorsal physis (not shown), left and right NCSs, physis of the dens, cranial and caudal physes. The dens scoring category included both the portion between the dens and the SOC cranially in C2 (pink lines), and the portions between the dens and the neural arches (pink and yellow lines), also known as the neuro‐dental synchondroses. The NCS scoring category included the growth cartilage on the left and right sides of the cranial SOC (turquoise and orange lines), whereas the cranial physis of the vertebral body (green lines) was scored on its own, without any portions of NCS. The caudal physis category included the caudal portions of the NCS (blue and purple lines) as for C3–C7, above. Note that the NCS makes a laterally pointing V‐shaped deviation midway between cranial and caudal. (F) 3D volume‐rendered model from C4, same colour codes as (A, B). The figure shows parts of some of the physes in a volume‐rendered model, and a rotating movie of the model is available in Video S1.

Closure was scored using three reconstructed CT planes. Scoring all hypoattenuating lines with different orientations in all parts of vertebrae separately resulted in over 70 scores per cervical spine. It was therefore decided to group some physes and portions of physes together by similar anatomical orientation for scoring, resulting in a total of 34 physeal scoring categories per cervical spine:

- In C1, three physes were scored: the (mid‐)dorsal physis, left and right NCSs (Figure 2A).
- In C3–C7, five physes were scored: the dorsal physis, left and right NCSs, and cranial and caudal physes of the vertebral body (Figure 2B–D). The NCS scoring category contained only the middle ~50% of the NCS, whereas the cranial and caudal portions of the NCS were grouped with the respective cranial and caudal physes of the vertebral body for scoring (Figure 2B,C).
- In C2, six physes were scored: the dorsal physis, left and right NCSs, physis of the dens, cranial and caudal physes of the vertebral body (Figure 2E). The dens scoring category included both the portion between the dens and the secondary ossification centre cranially in C2 and the portions between the dens and the neural arches, also known as the neuro‐dental synchondroses (Figure 2E). The NCS scoring category included the growth cartilage on the left and right sides of the cranial secondary ossification centre, whereas the cranial physis of the vertebral body was scored on its own, without any portions of NCS (Figure 2E). The grouping caudally in C2 was identical to C3–C7, above.

A 3D‐rendered model of C4 is shown in Figure 2F and rotated in Video S1. A hypoattenuating line was visible (Figure 2D) where the lateral portion fused with the medial portion of the primary ossification centre of the neural arch ventral to the transverse foramen. Closure of this fusion line was registered, but not reported due to unknown significance.

A scale was developed for scoring physeal closure in CT scans (Table 1). Scores 12–7 described the formation of ossification nuclei, ossification centres and physes, whereas Scores 6–0 described physeal closure (Figure S1). Three scores from 6: wide‐open, via 5: mid‐open, to 4: thin‐open were needed to reflect the fact that the dens, cranial and caudal physes of C2 were open to different extents.^15^ Scores 3 and 2 represented physes characterised as ‘closing’ by focal or wide bone bridging, respectively. Scores 1 and 0 represented physes characterised as ‘closed’, with or without sclerotic scars.^15^

**TABLE 1:** Scoring of physeal closure in computed tomographic scans.

Score	Observation	Short description	Interpretation/characterisation	Hertsch radiographic score^[15]^
12	No ossification nucleus either side of future physis	Cartilage model	Pre‐formation	N/a
11	Ossification nucleus^a^ one side of future physis	Ossification nucleus	Forming	N/a
10	Ossification centre^b^ one side of future physis	Ossification centre	Forming	N/a
9	Ossification nucleus both sides of future physis	Ossification nuclei	Forming	N/a
8	Ossification nucleus one side and ossification centre one side of future physis	Ossification nucleus and centre	Forming	N/a
7	Ossification centres both sides of wide, hypoattenuating physis‐like area; biconcave shape	Ossification centres	Forming	N/a
6	Hypoattenuating, linear area/physis, ≥3‐mm thick, rounded peripheral corners	Wide‐open	Open	Type A: wide‐open
5	Physis, ~2 mm thick, moderately rounded corners	Mid‐open	Open	N/a
4	Physis, ~1 mm thick, slightly rounded corners	Thin‐open	Open	Type B: thin‐open
3	Physis with focal bone bridges centrally or peripherally, visible in ≥2 planes of section	Focal bridging	Closing	N/a
2	Physis with wide bone bridge centrally, intermediate sclerotic scarring and thin, hypoattenuating line peripherally	Wide bridging	Closing	Types C: focal, central bridge, D: wider central and dorsal bridge, E: central and dorsal bridge complete, still open ventrally
1	Linear sclerotic scar, no hypoattenuating line left	Scar	Closed	Type F: sclerotic scar
0	Scar remodelled to medullary bone, no sclerosis left	Gone	Closed	Type G: gone/remodelled

Abbreviation: N/a, Not applicable.

^a^ Ossification nucleus: small, mineralised focus with generic spherical shape.

^b^ Ossification centre: larger mineralised focus with a shape resembling the mature bone.

On direct comparison, partial volume artefact was more often suspected of mimicking focal bone bridging in the bone kernel than soft tissue kernel reconstructed scans. With respect to developmental disease, it was considered a greater risk to underestimate than overestimate closure ages, thus all scoring was performed in soft tissue kernel reconstructed scans.

## 3 RESULTS

### 3.1 Population

The 35 cases comprised 13 foetuses (age range from 153 to 335 days of gestation), including one set of twins and 22 foals (age range from 0 to 438 days) with an age range from 153 days of gestation to 438 days old (Table S1). Age was not recorded for three foetuses. There were four premature and three dysmature cases. Sex distribution was 21 males, 12 females and 2 cases with sex unrecorded, and breed distribution was 12 Warmblood and 10 Standardbred horses, with seven miscellaneous ponies and six other horse breeds, respectively. Seventeen cases had systemic conditions, whereas 14 cases had orthopaedic conditions, including case 10 with torticollis (Table S1).

### 3.2 Factors affecting evaluation

The assessment of factors affecting evaluation is available in Table S2. C7 was accidentally dissected off in Case 27d and outside the collimation in Case 29, thus there were 243 rather than 245 completely included vertebrae. There were asymmetries ventrally on C6–C7 of Case 25, and C6 of Case 35 (Table S2).

### 3.3 Vertebral length

**FIGURE 3:**
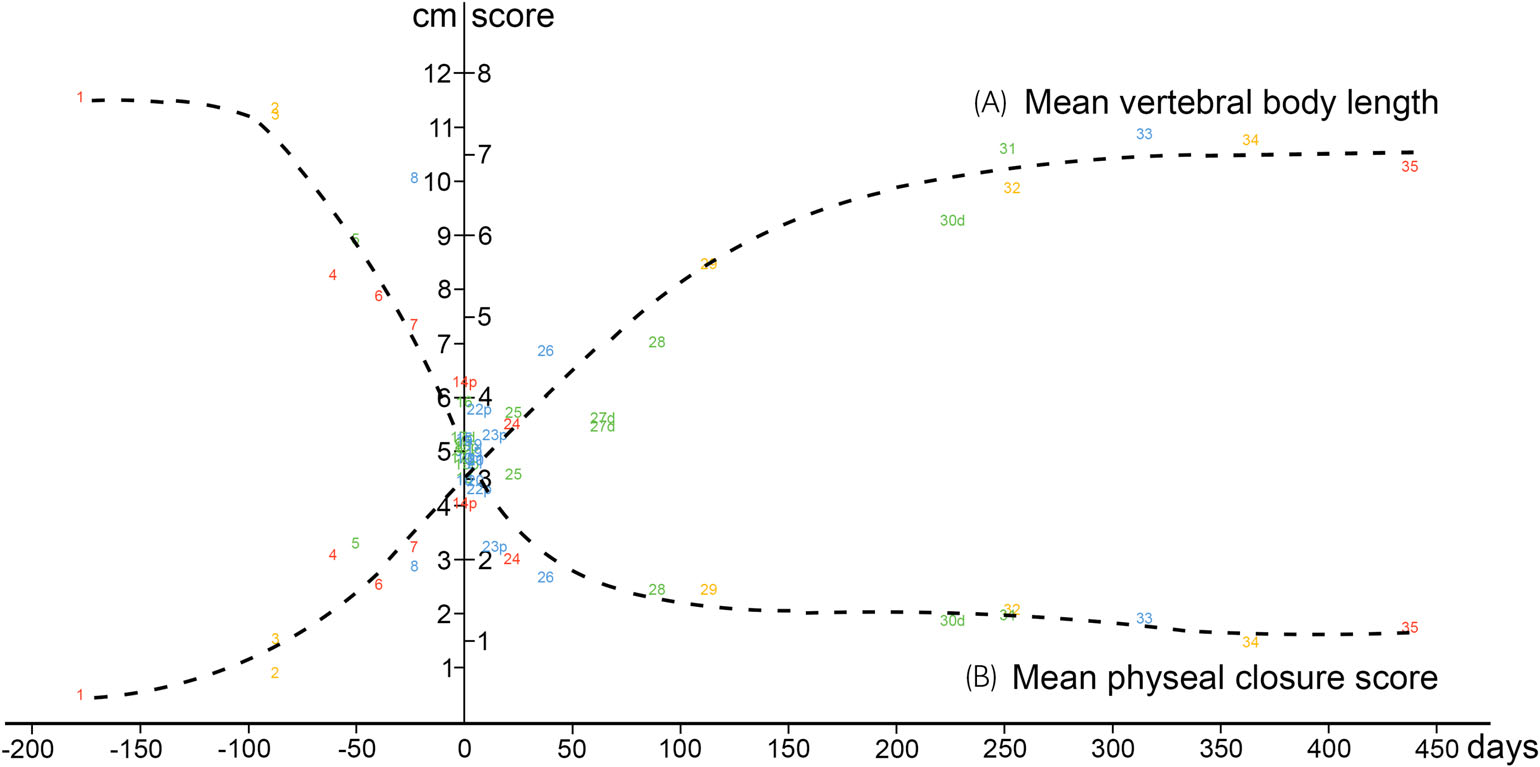
Mean vertebral body length (A) increased on a shallow sigmoid curve, whereas mean physeal closure score (B) decreased on a slightly steeper sigmoid curve, with age.

The length of all 243 vertebrae is available in Table S3, where cases were ordered by increasing mean vertebral length. Mean vertebral length increased on a shallow sigmoid curve with age (Figure 3A).

### 3.4 Radiological maturity—Early group

Qualitatively, the 35 cases fell into an early, middle and late group.

**FIGURE 4:**
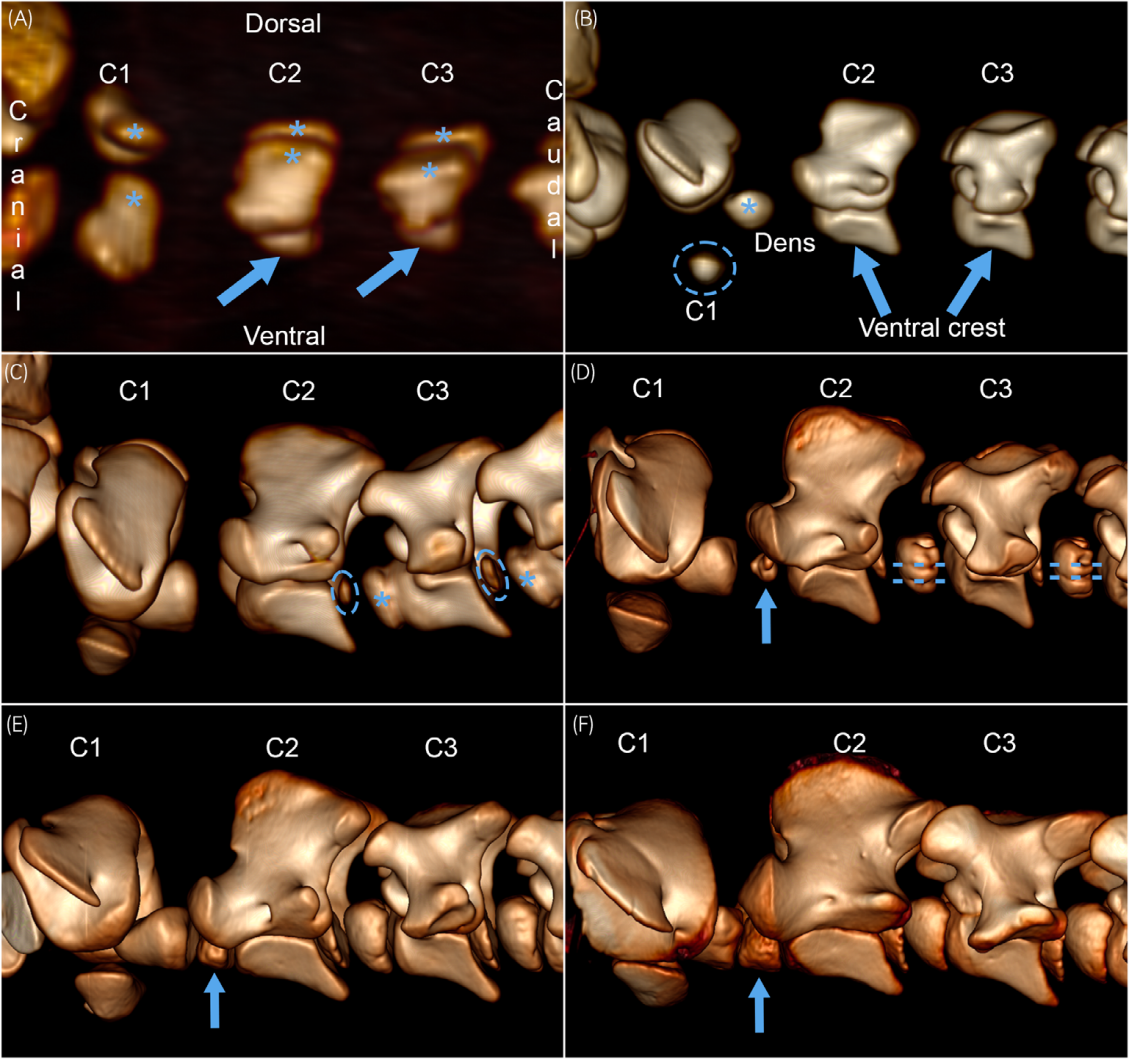
Radiological maturity, early group. 3D volume‐rendered CT images of the C1–C2–C3, left lateral views. (A) Case 1, 153 days of gestation. There are large primary ossification centres (asterisks) in the neural arches of C1 (slightly rotated) to C3, and smaller primary centres (arrows) in the bodies of C2–C3, but not C1. (B) Case 3, 244 days of gestation. In addition to the centres in (A), there is a large ossification centre in the dens (asterisk) and a small ossification centre in the ventral arch of C1 (dashed circle). The ventral crest (arrows) is present on the primary ossification centre of the vertebral bodies of C2–C3. (C) Case 6, 289 days of gestation. In addition to the centres in (A, B), there are large secondary centres (asterisks) cranially and smaller ossification nuclei (dashed circles) caudally in C2–C3. Unlike Cases 4–8 and 14p from 271 to 320 days of gestation, there is no secondary centre cranially in C2 and the cranial secondary centres are fused to the primary centres in the bodies of C3–C4. (D) Case 5, 280 days of gestation. There is a small secondary ossification centre (arrow) cranially in C2. There are regularly spaced, craniocaudally oriented grooves (dashed lines; likely vascular grooves) in the contours of the secondary centres cranially in C3–C4. (E) Case 4, 271 days of gestation. There is a properly formed secondary ossification centre cranially in C2 (arrow), and the secondary ossification centres cranially in C3–C4 are smoother than in Case 5 (D). (F) Case 14p, 0 days/16 h old, but 12 days premature, equivalent to 320 days of gestation. The secondary ossification centre cranially in C2 is almost as large and smooth as the ossification centres cranially in C3–C4.

The early group (Table S4A) comprised Cases 1–8 and 14p, equivalent to up to 320 days of gestation, united by the fact that some ossification centres were absent or incompletely formed. Radiological maturity did not increase in the exact same order as age (Table S4A). More details are available in Figure 4 and Table S4A. Ossification centres were interpreted to form in the following order: the primary centres in the neural arches of C1–C7, the primary centres in the vertebral bodies of C2–C7, the C2 dens ossification centre, the primary ossification centre in the ventral arch of C1, the secondary centres cranially in C3–C7, the secondary centres caudally in C2–C7 and finally, the secondary centre cranially in C2. Case 6 at 289 days of gestation (Figure 4C) was different from Cases 4–8 and 14p from 271 to 320 days of gestation (Figure 4D–F) in that there was no secondary centre cranially in C2, and the cranial secondary centres were fused to the primary centres in the bodies of C3–C6, thus Case 6 may represent premature closure. The transverse and articular processes appeared as direct extensions of the primary ossification centres of the neural arches, and the ventral crest appeared on the primary centre of the vertebral bodies (Figure 4A–C). It was noted that the dorsal physis zig‐zagged from left to right in mature cases and that the otherwise relatively straight profile of the NCS made a dorso‐laterally pointing V‐shaped deviation midway between its cranial and caudal ends (Figure 2C,E). In transverse slices, the NCS was variably straight and aligned with the dorsal plane (Figure 2A), or axio‐dorsally to ventro‐laterally obliquely oriented (Figure 2D).

### 3.5 Middle group

**FIGURE 5:**
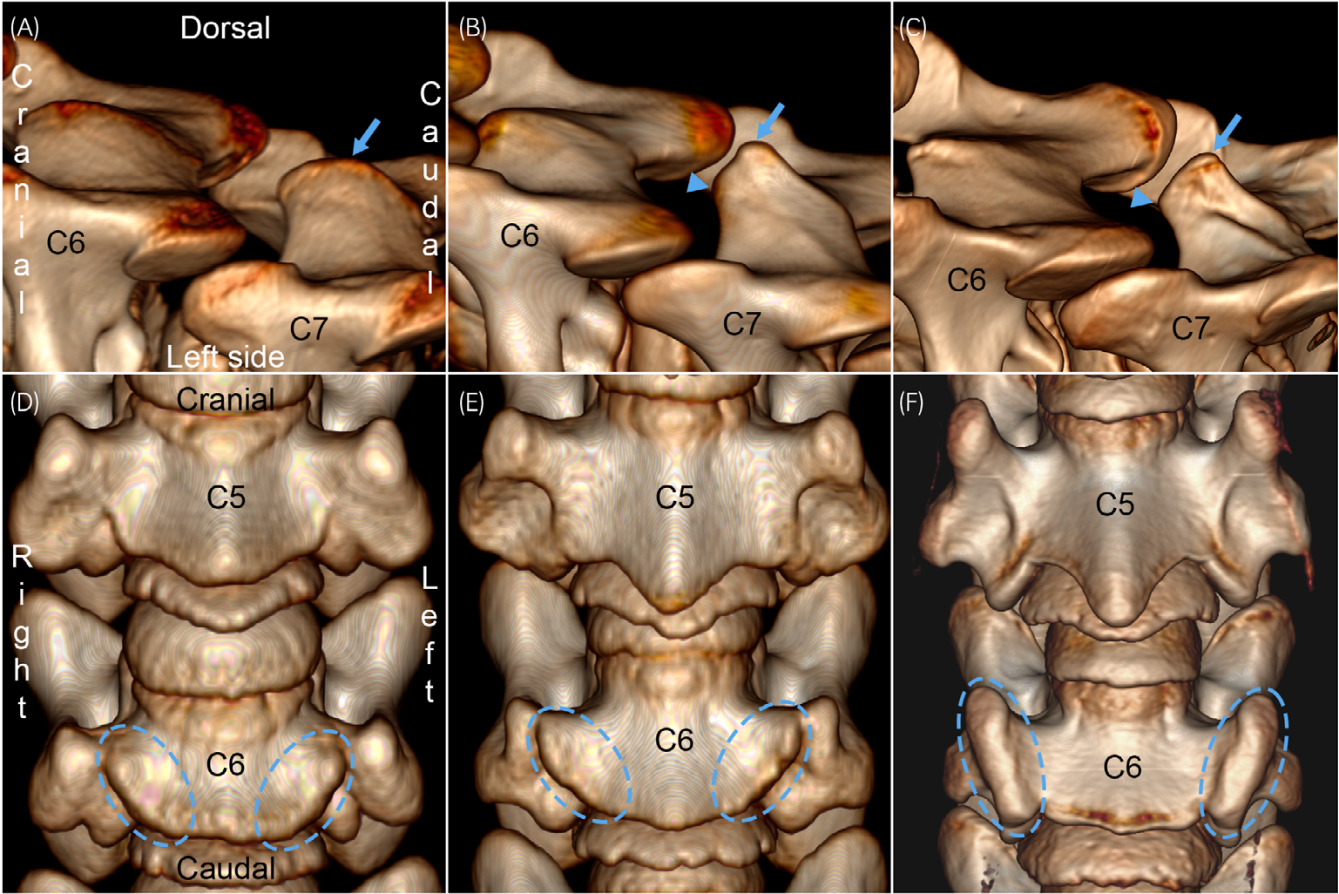
Radiological maturity, middle group. 3D volume‐rendered CT images of C6–C7 (left dorsolateral view) and C5–C6 (ventral view). (A) Case 16, 0 days/19 h. The dorsal spinous process (arrow) is only slightly taller on C7 than C6. (B) Case 22p, 6 days. There is a protruding focus (arrow) on the dorsal spinous process of C7, giving it a triangular appearance. The cranial border (arrowhead) of the neural arch is relatively low and round. (C) Case 25, 21 days. There is extra bone lipping (thickening) towards the dorsal margin (arrow), and the cranial contour (arrowhead) is taller and squarer, giving the dorsal spinous process a rectangular, mature shape. (D) Case 21, 6 days. The lamina region (dashed circles) protrudes only slightly further ventrally (between arrows) on C6 than C5. (E) Case 13, age not recorded. There are taller ridges (dashed circles) ventrally on C6 and they have bone lipping along part of their margins. (F) Case 24, 20 days old. There are large, mature‐shaped tubercles (dashed circles) ventrally on C6 with bone lipping along all of their margins.

The middle group (Table S4B) comprised Cases 9–13 and 15–25 from 327 days of gestation to 3 weeks old and were united by the fact that all major ossification centres had formed, and some physes were visibly starting to close in 3D‐rendered CT images. Again, radiological maturity did not increase in the exact same order as age and features (Table S4B) were also variably mature within cases. Notably, the dorsal physis was starting to close and the dorsal spinous process (Figure 5A–C) appeared as an extension of the fused primary ossification centres of the arch of C7 in the most mature cases. The larger, cranial part of the ventral laminae (Figure 5D–F) appeared as extensions of the primary ossification centre of the body of C6.

### 3.6 Late group

**FIGURE 6:**
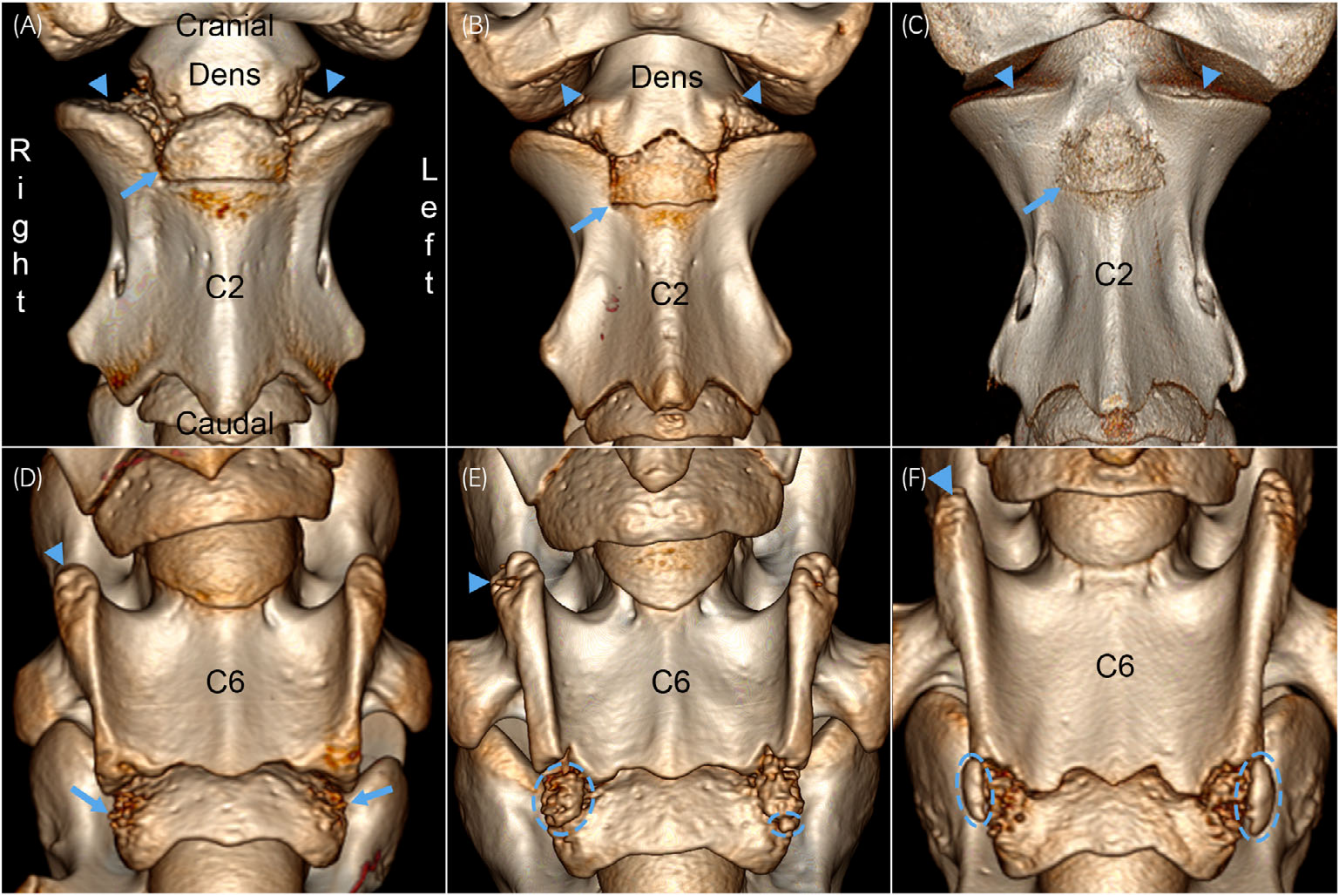
Radiological maturity, late group. 3D volume‐rendered CT images of C2 and C6 (ventral views). (A) Case 28, 93 days. The cranial ossification fronts of the neural arches in C2 (arrowheads), caudal ossification fronts of the neural arches in C1, and lateral ossification fronts of the dens have generalised dimples and tubes. The secondary centre cranially in C2 (arrow) looks like a regular ossification centre. (B) Case 29, 115 days. The caudal ossification fronts of the neural arches in C1 (arrowheads) and cranial ossification fronts of the neural arches in C2 have central dimples and tubes, whereas the dens only has dimples in small, peripheral regions. The cranial secondary ossification centre (arrow) is a square shape, outlined by linear grooves, in the ventral contour of C2. (C) Case 32, 260 days. The cranial ossification fronts of the neural arches of C2 have dimples in a small, peripheral region (arrowheads), whereas the other ossification fronts are smooth. The entire margin of the ossification centre cranially in C2 (arrow) is granular in appearance. (D) Case 29, 115 days. The cranial tips of the ventral laminae (arrowhead) have shallow dimples and short tubes. The caudal physis of C6 has dimples and tubes, more prominent in the ossification front of the secondary (arrows) than the primary ossification centre. (E) Case 30d, 227 days. The cranial tips of the ventral laminae have deep dimples and tall tubes (arrowhead). The caudal physis of C6 has mineralised bodies, some of which appear to have coalesced to form large, irregular‐shaped mineralised bodies (dashed circles) within the physis. (F) Case 34, 366 days. The cranial tips of the ventral laminae have deep dimples and a tall tube (arrowhead). The caudal physis has large, smooth and regular ellipsoid‐shaped mineralised bodies bilaterally (dashed circles). The ellipsoid bodies are located just lateral to the physis, at the junction with the growth cartilage of the caudal part of the ventral laminae, thus probably represent the secondary ossification centres for this part of the laminae.

The late group (Table S4C) comprised Cases 26–35 from 38 to 438 days old, united by the fact that all structures present were in the process of maturing. With the exception of Cases 27d and 28, radiological maturity increased in the same order as age (Table S4C). Certain features changed more dramatically than others, as follows:

- Specific ossification fronts of C2, C1 and the dens ossification centre (Figure 6A–C) progressed through stages of having generalised dimples and tubes, via having dimples and tubes in smaller central or peripheral regions, to eventual complete smoothness.
- The secondary centre cranially in C2 progressed from looking like a regular ossification centre (Figure 6A) to becoming a square shape outlined by linear grooves in the ventral contour of C2 (Figure 6B). With age, parts of the groove margins became granular in appearance and eventually, the entire margin was either granular or merged with the rest of C2 (Figure 6C).
- Cranial and caudal tips of transverse processes and ventral laminae progressed through similar changes, registered systematically for C5 in Table S4C and shown for the cranial part of the ventral laminae of C6 in Figure 6D,E.
- Although other physes showed more modest, similar changes, the caudal physis of the vertebral body of C6 (Figure 6D–F) changed quite dramatically. Initially, it progressed from being smooth to having dimples and tubes, more prominently so in the ossification front of the secondary than the primary ossification centre (Figure 6D). In more mature cases, the caudal physis of C6 additionally contained small mineralised bodies that sometimes appeared to have coalesced with each other and ossification front tubes to form large, irregular‐shaped mineralised bodies within the physis (Figure 6E). Cases 34 and 35 had even larger, smoother and more regularly ellipsoid‐shaped mineralised bodies bilaterally (Figure 6F) or right unilaterally, respectively. The ellipsoid bodies were located just lateral to the physis, at the junction with the growth cartilage of the smaller, caudal part of the ventral laminae, and probably represented the secondary ossification centres for this part of the laminae.

### 3.7 Physeal closure

As C7 was missing from Cases 27d and 29, there were 1180 available scores, listed in order of decreasing mean physeal closure score per case in Table S5. Mean physeal closure score decreased on a slightly steeper sigmoid curve than vertebral length, with increasing age (Figure 3B). Two cases appeared to represent outliers: Case 6 at 289 days of gestation (early group) scored more closed, and Case 27d at 65 days old scored more open than similarly aged cases. Disregarding Cases 6 and 27d, physeal closure occurred as follows:

- In C1, the left and right NCSs were closed in all cases from 115 days of age, whereas the dorsal physis was only closed in the oldest case at 438 days old.
- In C2–C7, the dorsal physis closed first and was consistently closed in all cases from 14 days of age; in C7, it even closed slightly earlier from 0 days old. The mid‐portion of the NCS in C2–C7 closed second and was closed in all cases from 38 days old. The physis of the dens in C2 closed third and was closed in all cases from 227 days old.
- The cranial physis of the vertebral body (including the cranial portion of the NCS) scored consistently more closed than the caudal physis of the vertebral body (and caudal portion of the NCS) and was therefore interpreted to be closing fourth, whereas the caudal physis was interpreted to be closing fifth and last of the scored physes.
- The cranial physis was closed in C7 of three cases: Case 23p at 14 days old, Case 28 at 93 days old and Case 35 at 438 days old. Other than these three cases, the cranial physis was not closed (i.e., Scores 1 and 0) in any case or in any vertebra, but it was consistently closing (Scores 3 and 2) in C3–C7 of all cases from 38 days old, and in C2 of all cases from 316 days old.
- The caudal physis was not closed (i.e., Scores 1 and 0) in any case, but it was consistently closing (Scores 3 and 2) in C3 and C5–C7 of all cases from 38 days old, and in C2 and C4 of all cases from 227 days old.

## 4 DISCUSSION

To the best of our knowledge, the current study is the first systematic description of the NCS in the cervical spine of horses. As CT is needed to visualise the NCS,^4^ the literature was searched for ‘foal spine CT’. This returned 56 papers, only one of which considered the NCS; Maierl et al.^4^ in C1–C2, and the NCS was also mentioned in a textbook by Wissdorf et al.^5^ However, when the search was expanded to include radiography, all papers referred back to only four sources containing raw data on physeal closure in the spine: Hertsch and El‐Salam Ragab (1977),^15^ Whitwell and Dyson (1987),^16^ Maierl et al. (1998)^4^ and the Butler et al. radiology textbook,^17^ thus more data are needed on physeal closure in general, as well as of the NCS and with advanced imaging.

### 4.1 Hospital population

It could be argued that studies of physeal closure should be carried out in normal foals. However, when a hypoattenuating line is described in CT scans for the first time and claimed to represent a physis, histological validation is mandatory. Some hospital cases have normal skeletons, and as long as this is the case, sacrificing healthy foals for validation is unjustified. The current population, therefore, represented convenience sampling of cases recruited via hospitals. With reference to previous studies in limbs,^10^, ^18^ the plan was to collect 10 necks, but it proved possible to collect 35 necks. Only time will tell if the reported closure ages are valid for non‐hospital cases; in the meantime, the current CT scans can be used to select cases for magnetic resonance imaging of the blood supply to the NCS, and for histological validation of suspected lesions. Although it was challenging to see physes in the twisted 3D model of torticollis Case 10, the deformity did not interfere with scoring of physeal closure in multi‐planar 2D slices. Premature closure undoubtedly occurs in some settings, but was only suspected cranially in C3–C6 of Case 6; a Shetland pony, and cranially in C7 of Cases 23p (Warmblood horse), 28 (Standardbred horse) and 35 (Fjord pony). In the Shetland pony, it was difficult to know whether premature closure reflected disease, or normal development for this chondrodystrophic breed.^19^ Children who survive chronic illness grow to smaller stature than their peers, but this is due to reduced cell division while physes are open, rather than premature closure.^20^ It should not be assumed that pre‐/dysmature and sick cases must be excluded because this affects closure, rather: exclusion should be based on evidence the literature does not contain yet.^4^, ^15^, ^16^, ^17^ Variably mature and sick cases were included because we consider this study the start of a database from which it will become possible to plot growth curves stratified by breed, sex, maturity and disease status in future.

### 4.2 Reporting physeal closure

When reporting physeal closure, it must be tied to some trait of the individual that was scored. Although cases were collected prospectively, gathering all the desired information proved challenging. Data were missing for a wide range of reasons, including that the dam of Case 6 was at pasture with the stallion, thus exact covering date was unknown. The most common way to report physeal closure is tied to the post‐natal age of the case.^4^, ^15^, ^16^, ^17^ However, foals have been born live from 294 to 419 days of gestation.^21^ In the current live foals for which it was known, gestation length ranged from 312 to 367 days (Table S1); a difference of 55 days. The dorsal and NCS physes were closed in most cervical vertebrae by 38 days of age. If these physes were closed in a newborn foal and only post‐natal age was known, it could lead to the erroneous conclusion that they were prematurely closed if the gestation length was more than 38 days longer than normal. Knowing gestation length was therefore particularly important for the current study where variably mature cases were deliberately included, but maturation issues such as incomplete ossification of cuboidal bones may be present without detectable surface signs,^22^ thus both gestation length and post‐natal age should be registered to avoid confounding error in all reports of physeal closure. Initially, vertebral length was measured because crown‐rump length could not be obtained for some cases as only the neck was received, but it was also considered advantageous to have an objective measure of size from the CT scan itself. The current population includes 11 breeds of horse with different newborn and adult sizes. Even when stratifying by breed, it was not possible to place cases in perfectly agreeing age‐ or vertebral length order because the largest breed groups also contained the most pre‐ and dysmature cases. This was the reason for electing to present all breeds together, and for including Figure 3 to show that length and closure fell on relatively smooth curves, even if it was challenging to place the cases in any kind of logical order. We could have measured many more vertebral dimensions manually, but algorithms are now available that segment and analyse bones automatically, which will do a more precise job in future. As discussed, physeal closure could not be tied to vertebral length in the current population, but it was nonetheless reported to allow for informal comparison at our own and other hospitals in the interim before automated comparison becomes possible. The radiological maturity of each case was also evaluated, but we never intended for physeal closure to be tied to as qualitative a measure as radiological maturity. Rather, maturity was evaluated because it is considered part of standard radiology reporting and because the evaluation will help distinguish between normal development and lesions in future. In sum, the ideal way to present physeal closure is probably linked to gestation length and post‐natal age, labelled with whether the case showed any signs of pre‐ or dysmaturity at birth. We will strive to achieve this in future, but for the current cases, we resigned to presenting physeal closure tied to post‐natal age because this seemed the best option, and may enable some comparison with literature (Tables 1 and 2).^4^, ^15^, ^16^, ^17^

**TABLE 2:** Comparison of the current results with existing literature; age translated from days into months.

Reference^a^	Cases, age range, breed, population	Distribution of relevant cases	Modality	Relevant scoring grades	C1 NCS	C1 mid‐dorsal	C2^b^ NCS	C2 dens physis	C2 cranial physis	C2 caudal physis	C3–C7 cranial physes	C3–C7 caudal physes
Current results	35 cases 5 months of gestation 14.4 months old Misc. breeds Hospital population	1–180 days old: 16 cases 6–12 months old: 4 cases 1–2 years: 2 cases	CT	Score 1: complete scar Score 0.5: partial scar Score 0: gone	Scar ≥3.8 months Partial scar ≥8.3 months Gone ≥8.5–14.4 months	Scar at 14.4 months	Scar ≥1.2–14.4 months	Scar ≥7.4–14.4 months	Scar ≥10.4–14.4 months	Bone bridges but not closed	Bone bridges but not closed (three exceptions)	Bone bridges but not closed
Maierl et al. (1998)^4^	32 cases—Newborn‐5 years old Misc. breeds Hospital, slaughterhouse	Information missing	Radiography 32 cases CT ≥1 case, 6 months old Macroscopic anatomy 27 cases	Just descriptive: starts closing, scar, closed, gone	Macroscopic ‘scar’: at about 6 months old Gone: in yearlings	Macroscopic and radiographic scar: 12–14 months old	Macroscopic closed: 2–4 months old	Macroscopic closed: 12 months old Radiographic scar 12–13 months	Macroscopic starts closing: by 36 months old Radiographic scar: 36 months Gone: 48–60 months old	Gone: 48–60 months old	N/a	N/a
Hertsch and El‐Salam Ragab (1977)^15^	140 cases 1 day–8 years old Warmblood horses 8 dead newborn foals, rest: no information	1–180 days old: 18 cases 6–12 months old: 18 cases 1–2 years old: 20 cases	Radiography	See Table 1 (Score E: partial scar) Score F: complete scar Score G: gone	N/a	N/a	N/a	Scar: 7–9 months old Gone: 10–12 months old	Scar: during 13–36 months Gone: during 37–48 months	Scar: from start of 49–60 months Gone: from 61 months	Scar: during 25–48 months Gone: from start of 49–60 months	Scar: from start of 49–60 months Gone: from 61 months
Whitwell and Dyson (1987)^16^ and Butler et al. (2016)^17^	Information missing (many Thoroughbreds)	Information missing	Radiography	Just descriptive: complete	N/a	(Faint line in neonate)	N/a	Approximately 7 months	No information	Complete 48–60 months	Complete by 24 months	Complete by 48–60 months
Comparison					Current CT scar gone 3.5 months later than Maierl macroscopic scar	Current CT scar gone 0.4 months later than Maierl macroscopic scar	Current CT scar appears 0.8 months earlier than Maierl macroscopic scar	Current CT scar agrees with Hertsch radiographic scar, Maierl later than Hertsch radiographic scar	Current CT scar 2.6 months earlier than Hertsch/25.6 months earlier than Maierl, Hertsch/Maierl just overlap	Hertsch and Maierl overlap	Whitwell complete 1 month earlier than Hertsch scar	Whitwell complete 1 month earlier than Hertsch scar

Abbreviations: CT, computed tomography; NCS, neuro‐central synchondroses; N/a, not applicable.

^a^ Wording varies slightly to be as close to the original citation as possible.

^b^ C2 has a dorsal physis that closed before the NCS, but it is not included in the table as there were no data available for comparison.

### 4.3 Grouping of physes for scoring

If the cranial, middle and caudal portions of the left and right NCS were scored separately, there would have been 6 × 7 = 42 scores for the NCS alone. Until we know more about where lesions occur,^9^ it is difficult to say if we need to know closure times separately for the different portions of the NCS. Conversely, there are already clinical examples where we need to know when the NCS closes relative to other vertebral physes.^23^ We therefore elected to limit NCS scoring categories somewhat by grouping portions of the NCS as described and instead expand the scoring to include all physes that appeared in addition to the NCS during the sampled age interval.

### 4.4 How do equine cervical vertebrae grow?

The most important aspect of the current results is the story they tell about how equine cervical vertebrae grow.

#### 4.4.1 Equine vertebrae matured in distinct early and late phases

There was no prior information about when the NCS was present in C3–C7 of horses,^4^ thus, the current upper sampling age was chosen arbitrarily. However, radiographic studies confirm that vertebral growth is not complete until the horse is 5 years old.^4^, ^15^, ^16^, ^17^ Comparison must be done with care as there are notable species differences, but the present results tell a similar story as in children, where vertebral maturation occurs in a distinct pre‐teen, early phase and later teenage phase.^24^ In children, the primary ossification centres of the neural arches and vertebral bodies are present at birth, and the dorsal and NCS physes close before 12 years of age.^24^ The human counterparts of the secondary centres cranially and caudally in the vertebral bodies; the ‘ring apophyses’ and secondary centres in the spinous (dorsal), articular and transverse processes appear in teenagers and close by the age of 25 years old.^24^ In current cases 1–33 up to 316 days old, the primary ossification centres of the arches and bodies appeared, the secondary centres cranially and caudally in the bodies also appeared, and the dorsal and NCS physes closed,^4^ thus these cases seem to capture the equine equivalent of the early, pre‐teen phase of vertebral maturation.^24^ Small secondary centres were present in the caudal part of the ventral laminae of C6 in the oldest Cases 34 and 35,^16^ suggesting that the late maturation phase^24^ starts around 366–438 days or 12–14 months of age. The clinical relevance of the fact that some parts of vertebrae completely finish their maturation during the early phase, while others have barely started^24^ is discussed further below.

#### 4.4.2 Vertebral processes and laminae appeared on different ossification centres in different ways

The articular processes, transverse processes, dorsal spinous process and cranial, large portion of the ventral laminae of C6 appeared as extensions of two different primary ossification centres on different timescales (Tables S4A and S4B). The small, caudal portion of the ventral laminae ossified from its own secondary centre,^16^ rather than as an extension of an existing centre, and its physis was continuous with the caudal physis of the body of C6 (Figure 6F). The fact that processes and laminae ossify in different ways should probably be considered when discussing how they suffer asymmetry or disease.

#### 4.4.3 All portions of the NCS contributed to growth in all directions

Once ossification centres appeared, it was easier to appreciate the orientation and shape of the different physes, including notched and zig‐zagged profiles. Material from the current cases is being decalcified for histology, but in the meantime, the equine CT results can tentatively be combined with the porcine sections (Figure 1C,D),^1^ where the largest mid‐portion of the NCS contained proliferative zones producing cells in the dorsal and ventral directions, thus contributing to height‐wise growth of the neural arches and vertebral body. However, as the mid‐portion of the NCS was oblique in the transverse plane (Figure 2D), proliferation would also produce cells in the axial and lateral directions, contributing to width‐wise growth. Finally, there was a small V‐shaped notch in the middle of the NCS (Figure 2C,E), where proliferation would produce cells in the cranial and caudal directions contributing to length‐wise growth, and we suspect the zig‐zagged profile of the dorsal physis served a similar purpose. The smaller cranial and caudal portions of the NCS were oblique in all three planes (Figure 2B–D) and therefore probably also contributed to growth in all directions. The different portions of the NCS likely make variable contributions to growth in different directions depending on factors like how oblique they are (compare Figures 2A and 2D), how thick their proliferative zones are (Figure 1C,D), and how long they remain open (Table S5). All portions of the NCS can therefore probably contribute to the growth of the arch and body in all directions, but they do so to different extents and on different timescales.

#### 4.4.4 Ossification fronts go through phases of more active growth at different ages

Ossification front dimples and tubes were known from earlier micro‐CT scans of equine limb samples.^14^ It has previously been validated that tubes are centred on patent cartilage canals, whereas dimples are centred on patent or chondrifying canals in histological sections.^14^ Chondrification is the physiological process by which the blood supply to growth cartilage regresses, and this occurs in an orderly fashion, typically from central to peripheral regions, at different ages for different limb joints.^10^, ^18^ The current tubes and dimples likely represent the same patent and chondrifying canals as in limb epiphyses.^14^ The main difference was that from birth, limb epiphyses only progressed from being irregular to becoming smooth,^14^ whereas some of the current evaluated ossification fronts (Figure 6D–F) progressed from initial smoothness to general irregularity after birth. Tubes and dimples probably signify periods of particularly active vascular‐dependent growth, and it is important to be aware that such periods also occur after birth, as they provide the dynamic backdrop against which the pathogenesis of diseases like osteochondrosis must be understood.

### 4.5 Relationship between the current results and disease

Although focal lesions will be described in a future report, it is necessary to comment on some aspects of the disease because they relate to and emphasise the importance of the current included results.

#### 4.5.1 Abnormal absence of ossification centres

Abnormal absence of a secondary ossification centre was suspected cranially in C2 of Case 6, and caudally in the left ventral lamina of C6 in Case 35. When premature cases were included in a recent study of distal tarsal osteoarthritis, changes were observed that generated the new working hypothesis that failure of a nutrient artery leads to delayed formation of the ossification centre and incomplete ossification of tarsal cuboidal bones.^22^ Similar nutrient artery failure could explain the absence of the ossification centre cranially in C2 of Case 6, but for Case 35, a second explanation must also be considered. Asymmetry is commonly observed in caudal cervical vertebrae, sometimes referred to as ‘transposition’ of the ventral laminae of C6.^19^, ^25^, ^26^, ^27^ Asymmetries range from simple absence of the caudal part of one ventral lamina,^25^, ^27^ to complex changes on C6–C7^19^, ^26^ (like current Case 25) and Th1.^25^ When changes are complex and span segments,^25^ they may represent some form of abnormality in the HOX gene family that regulates vertebral morphology changes at segment junctions.^19^, ^25^, ^26^ Conversely, when the only noted change is the absence of the caudal part of the ventral lamina of C6,^25^, ^27^ this could represent delayed or definitive non‐formation of the cartilage model and ossification centre for this part of the lamina due to vascular failure.^22^ The current results emphasise the need for precise diagnoses, especially distinguishing between whether asymmetry cases represent simple absence of an ossification centre or complex deformity at segment junctions, as these may have different pathogeneses, implications and management.

#### 4.5.2 How the NCS contributes to growth and hypotheses for stenosis

The case of Yang et al.^9^ had ischaemic chondronecrosis of the mid‐portion of the NCS, generating the first working hypothesis that: failure of the blood supply to the mid‐portion of the NCS leads to delayed growth, shortening and stenosis at the mid‐portion of the spinal canal. During the study, questions arose. The hypoattenuating line of the mid‐NCS quickly became very thin; thus, could growth cartilage not simply survive by diffusion from adjacent bone following vascular failure?^10^ If the NCS is known for its role in scoliosis,^6^, ^7^ why do horses commonly suffer stenosis without concurrent scoliosis? The porcine experimental studies confirm that scoliosis will only develop if certain criteria are fulfilled: young age, many fixation points and long duration,^6^, ^7^ thus, perhaps spontaneous equine lesions do not meet these criteria. The current results also open the possibility that scoliosis arising due to lesions in the mid‐NCS during the early phase^9^ is corrected through compensatory growth from the cranial and caudal portions of the NCS during the late phase of vertebral maturation (Section 4.4.1, above). If the cranial and caudal portions of the NCS are able to compensate in length, but not height because of differential contribution in the two directions (Section 4.4.3, above), scoliosis could resolve while stenosis persists. In 1993, Pool^28^ described that on post‐mortem examination of wobbler cases, stenosis was identified at the cranial and caudal ends of vertebrae and occurred because the neural arches were shortened at these sites. This generated the second working hypothesis that failure of the blood supply to the cranial/caudal portions of the NCS leads to delayed growth, shortening and stenosis at the cranial/caudal portions of the spinal canal. Both hypotheses probably occur, but comparison to limb osteochondrosis^10^ supports that because the cranial and caudal portions were thicker (Figure 2B,C,E) and open for longer than the mid‐portion of the NCS (Table S5), the second hypothesis is likely to occur and result in ischaemic chondronecrosis more frequently than the first. Again, the current results emphasise the need for precise diagnosis of the location of the stenosis: in the mid‐portion versus cranial/caudal ends of the spinal canal, as these could develop on different timescales and have different potential for compensatory growth.

#### 4.5.3 NCS growth interacting with facet joint osteoarthritis as a cause of stenosis

Osteochondrosis is the most common cause of juvenile osteoarthritis in facet joints and other joints of horses^8^, ^22^ and pigs.^1^ The literature contains studies variably concluding that facet joint osteochondrosis/osteoarthritis is associated with stenosis/malformation,^29^ is not associated with stenosis,^30^ or is associated with stenosis in some, but not other facet joints of the same horse.^8^, ^11^, ^31^ It is now possible to see a hypothesis that explains this: facet joints that are markedly enlarged can probably cause cord compression independent of other factors,^8^, ^29^ whereas facet joints that are only moderately enlarged are more likely to cause compression if the height of the neural arch is also reduced. The best‐documented way to reduce arch height is by tethering NCS growth,^7^ and a spontaneous lesion of ischaemic chondronecrosis known to cause delayed growth in limb physes was also identified in the NCS of one foal.^9^ Affected individuals usually have osteochondrosis in more than one site, and in the spine, potential sites include the facet joints,^7^, ^11^ NCS^9^ and intervertebral joints.^1^ Surgeons are increasingly able to remove facet joint OCD fragments,^23^ but one case developed (early/functional) stenotic myelopathy within 6 weeks of fragment removal^23^ and in limb joints, it is common to wait until the age threshold for osteochondrosis development in the stifle and hock joints have passed before removing any fetlock fragments. The current study provides information about when the NCS is susceptible to osteochondrosis, but we would like to discover how long it takes for spontaneous lesions^9^ to manifest as reduced arch height,^7^ as well as the age thresholds for osteochondrosis development in facet joints. The spinal cord also grows^32^ and compression will depend both on having a narrowed spinal canal and a cord that has grown thick enough to become compressed; thus, cord growth should be studied further as well.

## 5 CONCLUSIONS

The NCS was a thin physis that contributed mainly to height‐wise growth, but also width‐ and length‐wise growth of the vertebral body and neural arches. The mid‐NCS was closed in all cervical vertebrae from 115 days of age. The NCS warrants further investigation in the pathogenesis of equine cervical vertebral stenosis.

## FUNDING INFORMATION

This study was funded by grant number H‐20‐47‐553/NFR323877 from the Swedish–Norwegian Foundation for Equine Research/Research Council of Norway, with contributions from Norsk hestesenter and Jordbruksavtalen.

## CONFLICT OF INTEREST STATEMENT

The authors have declared no conflicting interests.

## AUTHOR CONTRIBUTIONS

**Kristin Olstad:** Funding acquisition; writing – original draft; conceptualization; methodology. **Mari Dahl Bugge:** Writing – review and editing; methodology; conceptualization. **Bjørnar Ytrehus:** Conceptualization; methodology; writing – review and editing. **Anne Selvén Kallerud:** Conceptualization; writing – review and editing; methodology; supervision.

## DATA INTEGRITY STATEMENT

Kristin Olstad had full access to all the data in the study and takes responsibility for the integrity of the data and the accuracy of the data analysis.

## ETHICAL ANIMAL RESEARCH

The study was approved by the institution's Ethical Committee for approval of studies with animal patients (Norwegian University of Life Sciences, approval number 14/04/04273‐69) and was in accordance with Norwegian legislation regarding the use of animals in research (FOR‐2017‐04‐05‐451).

## INFORMED CONSENT

Horse owners provided informed consent for the use of the cases for this study.

6

### PEER REVIEW

The peer review history for this article is available at https://www.webofscience.com/api/gateway/wos/peer-review/10.1111/evj.14093.

## Supporting information


**Figure S1.** Sample dorsal plane images if closure scores 6–0 in the neuro‐central synchondrosis (NCS) of C4. Scores pertain to the mid‐portion of the NCS (between dashed lines).

**Table S1.** Population.

**Table S2.** Assessment of factors potentially affecting radiological evaluation.

**Table S3.** Vertebral length (cm). Cases are presented in order of increasing mean vertebral length.

**Table S4A.** Radiological maturity—early group.

**Table S4B.** Middle group.

**Table S4C.** Late group.

**Table S5.** Physeal closure. Cases are presented in order of decreasing mean closure scores.

**Video S1.** Rotating movie of Figure 2F: C4 of Case 27d, please regulate playback speed using the play/pause slider.

## Data Availability

The data that support the findings of this study are available from the corresponding author upon reasonable request: Open sharing exemption granted by editor for this descriptive report.
