# Supplementary material for: Closure of the neuro‐central synchondrosis and other physes in foal cervical spines
Source: Equine Vet J. 2024 Apr 9;57(1):217–31. doi: 10.1111/evj.14093 (PMC11616957; doi:10.1111/evj.14093)
Supplement: Supplementary file 1 — Figure S1. Sample dorsal plane images if closure scores 6–0 in the neuro‐central synchondrosis (NCS) of C4. Scores pertain to the mid‐portion of the NCS (between dashed lines). [file EVJ-57-217-s004.pdf]

Equine Veterinary Journal BEVA

Figure S1: Sample dorsal plane CT images of closure scores 6-0 in the neuro-central synchondrosis (NCS) of C4. Scores pertain to the mid-portion of the NCS (between dashed lines).

CHARACTERISATION: OPEN

WIDE-OPEN

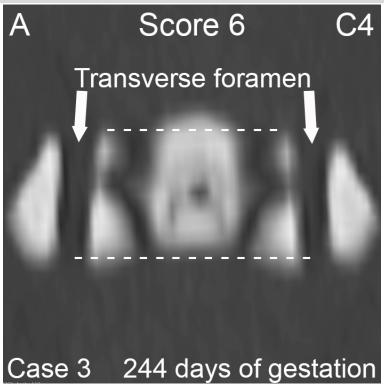

Hypoattenutating linear area/physis, ≥3 mm thick, rounded peripheral corners

MID-OPEN

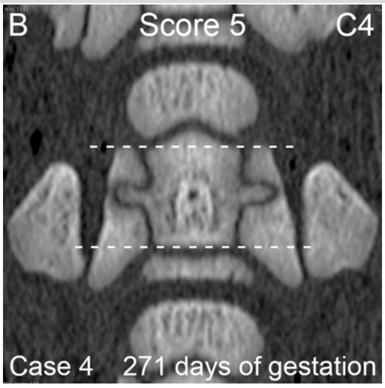

Physis, ~2 mm thick, moderately rounded corners

THIN-OPEN

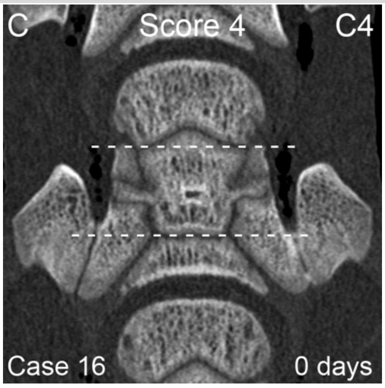

Physis, ~1 mm thick, slightly rounded corners

CHARACTERISATION: CLOSING

FOCAL BRIDGING

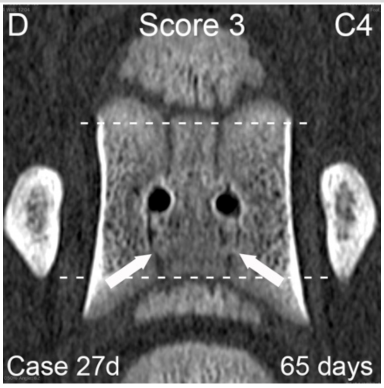

Physis with focal bone bridges centrally (arrows) or peripherally, visible in ≥2 planes of section

WIDE BRIDGING

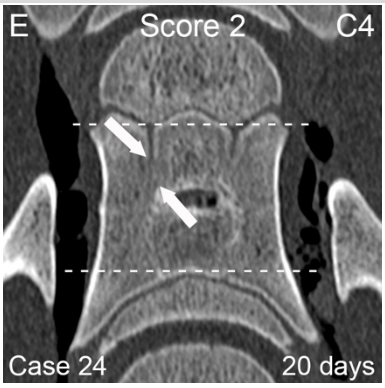

Physis with wide bone bridge centrally (between arrows), intermediate sclerotic scarring and thin, hypoattenuating line peripherally

CHARACTERISATION: CLOSED

SCAR

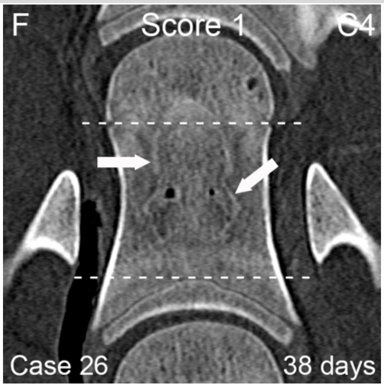

Linear sclerotic scar (arrows), no hypoattenuation left

GONE

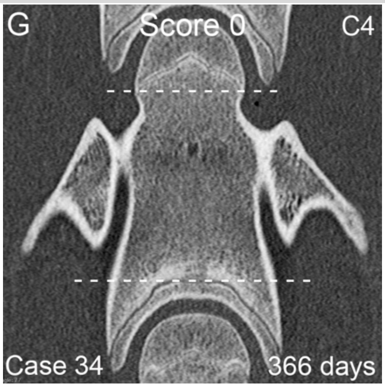

Scar remodelled to medullary bone, no sclerosis left
