# Supplementary material for: Closure of the neuro‐central synchondrosis and other physes in foal cervical spines
Source: Equine Vet J. 2024 Apr 9;57(1):217–31. doi: 10.1111/evj.14093 (PMC11616957; doi:10.1111/evj.14093)
Supplement: Supplementary file 2 — Table S1. Population. [file EVJ-57-217-s009.pdf]

Equine Veterinary Journal BEVA

TABLE S1: Population. Cases were numbered 1-35 by increasing age, or by increasing size if age was equal or unrecorded. Premature or dysmature cases were labelled 'p' or 'd', respectively. The number in the parentheses in column 2 is the gestation length.

Case[†]	Age, days[‡]	Maturity	CRL, cm	Weight, kg	Breed	Sex	Cause of death/euthanasia	Systemic condition; culture	Orthopaedic condition	Sample
1	153	N/a	30.7	0.5	Connemara	Male	Dam colic died, typhlitis	Endotoxaemia; N/d	No	Whole body
2	244	N/a	41.2	1.5	Arabian	Male	Twin, hydroallantois, induced	No; N/d	No	Whole body
3	244	N/a	62.2	7	Arabian	Male	Twin, hydroallantois, induced	No; N/d	No	Whole body
4	271	N/a	N/r	14	Likely Icelandic	Male	Unidentified	No; negative EHV1/4 + EVA	No	Dissected neck
5	280	N/a	85	N/r	Standardbred	N/r	Dam tumour, peritonitis	Endotoxaemia; N/d	No	Dissected neck
6	289	N/a	67.7	12.5	Shetland	Female	Dystocia, malposition	No; N/d	No	Whole body
7	310	N/a	83.3	14	Welsh Cob	Female	Dystocia, hydrocephalus	No; N/d	No	Whole body
8	311	N/a	80.2	18	Warmblood	Male	Unidentified	No; negative EHV1/4 + EVA	No	Whole body
9	327	N/a	110.4	48	Standardbred	Male	Dystocia, malpresentation	No; N/d	No	Whole body
10	335	N/a	N/r	42	Warmblood	Female	Dystocia, torticollis	No; N/d	Torticollis	Dissected neck
11	N/r	Term foal	95	N/r	Warmblood	Male	Unidentified	No; negative EHV1/4 EVA *Leptospira*	No	Whole body
12	N/r	Term foal	105	N/r	Warmblood	N/r	Dam trauma died, peritonitis	Endotoxaemia; negative EHV1/4 EVA	No	Dissected neck
13	N/r	Term foal	109.3	45.5	Coldbl. trotter	Female	Dystocia, breech	No; N/d	No	Whole body
14p	0 (320)	12 days premature	N/r	23	Welsh Mountain	Female	Congenital limb deformity	No; N/d	FLD: contracture	Dissected neck
15	0 (340)	Term foal	113.8	50.5	Warmblood	Male	Respiratory arrest (dam colic)	No; N/d	No	Whole body
16	0	Term foal	N/r	55	Standardbred	Female	Umbilical haemorrhage	No; N/d	No	Dissected neck
17d	0 (367)	35 days dysmature, HT	N/r	62	Standardbred	Female	Pulmonary oedema (dam colic)	Hypothyroid; N/d	Extensor #	Dissected neck
18p	1	Clinically premature	108.7	37.5	Standardbred	Female	Neonatal maladjustment	No; N/d	FLD: laxity	Whole body
19	3	N/r	N/r	45	Warmblood	Male	Rejected, pneumonia	Pneumonia; *E. coli, Klebsiella pneum.*	No	Intact neck
20	6	N/r	112.6	37	Warmblood	Female	Pneumonia, deformity	Pneumonia; N/d	FLD: laxity	Whole body
21	6	N/r	113.5	53.5	Warmblood	Male	Septic polyarthritis, anaesthetic death	Sepsis; negative (antibiotics)	Polyarthritis	Whole body
22p	6 (318)	14 days premature	N/r	73	Warmblood	Male	Bladder #, anaesthetic death	Uraemia; N/d	No	Intact neck
23p	14 (312)	20 days premature	130.0	65.5	Warmblood	Male	Septic polyarthritis, deformity	Sepsis; *A. equuli*	Polyarthritis, FLD: laxity	Whole body
24	20	N/r	N/r	N/r	Icelandic	Male	Pneumonia, kidney failure	Pneumonia; *S. dysgalactiae, S. aureus*	No	Dissected neck
25	21	N/r	N/r	78	Standardbred	Male	Dystocia, pressure necrosis	Wound; *E.coli* (antibiotics)	Hock lesion	Dissected neck
26	38	N/r	N/r	105	Warmblood	Male	Persistent ductus arteriosus	Lymphadenitis; *R. equi*	No	Intact neck
27d	65	Unknown dysmature, HT	N/r	53	Standardbred	Male	Hypothyroid, deformity	Hypothyroid; negative	Extensor #; FLD: contracture	Dissected neck
28	93	N/r	N/r	90	Standardbred	Female	Pneumonia, polysynovitis	Pneum: *E. coli*, *R. equi* negative, no PCR	Polysynovitis	Intact neck
29	115	N/r	N/r	170	Coldblooded	Female	Acute lame, # teres ligament	No; N/d	#teres ligament	Intact neck
30d	227 (367)	35 days dysmature	N/r	N/r	Standardbred	Male	Stiff gait, bilateral hip dysplasia	No; N/d but *R. equi* on pharynx histology	Hip dysplasia	Dissected neck
31	253	N/r	N/r	210	Standardbred	Male	Tricuspid endocarditis	No; negative, bacterially initially	No	Intact neck
32	260	N/r	N/r	249	Thoroughbred	Male	Colic, post-op adhesions	Endotoxaemia; N/d	No	Dissected neck
33	316	N/r	N/r	313	Warmblood	Male	# pelvis, historic pneumonia	Lungs: *S. zooep, M. spp.; R. equi* negative	# pelvis	Intact neck
34	366	N/r	N/r	N/r	Coldbl. trotter	Male	Head tumour: dental/sinus	No; N/d	No	Intact neck
35	438	N/r	N/r	250	Fjord	Female	Acute ataxia, neck pain	No; N/d	Neck pain	Intact neck

Abbreviations: CRL, Crown-rump length. FLD, Flexural deformity. HT, Hypothyroid. N/a, Not applicable. N/d, Not done. N/r, Not recorded. #, Rupture or fracture.
[†]p, Premature cases; d, Dysmature cases. [‡]Cases 1-13 were abortions and stillbirths: age is days of gestation. For cases born live, gestation length is given in parenthesis if known.
