# Supplementary material for: Closure of the neuro‐central synchondrosis and other physes in foal cervical spines
Source: Equine Vet J. 2024 Apr 9;57(1):217–31. doi: 10.1111/evj.14093 (PMC11616957; doi:10.1111/evj.14093)
Supplement: Supplementary file 3 — Table S2. Assessment of factors potentially affecting radiological evaluation. [file EVJ-57-217-s008.pdf]

Equine Veterinary Journal BEVA

Table S2: Assessment of factors potentially affecting radiological evaluation.

Case[†]	Decomposition	Dissection	Collimation	Dorso-ventral positioning	Latero-lateral positioning	Rotation	Artefacts	Asymmetry of the ventral laminae of C6
1	No	Not dissected	All in	Straight	Mild left bowing	C1 rotated right side ventral	No	No
2	Yes; large holes left side	Not dissected	All in	Dorsal bowing	Relatively straight	C1 rotated left side ventral, C7rotated right side ventral	No	No
3	No	Not dissected	All in	Dorsal bowing	Straight	C1 angled, not rotated	No	No
4	Yes, or normal development: tubular defects especially in cranial vertebral SOCs	Dissection ok	All in	Straight	Mild left bowing	C1 rotated left side ventral	No	No
5	Yes, or normal development: tubular defects especially in cranial vertebral SOCs	Dissection ok	All in	Straight	Straight	Straight	No	No
6	No	Not dissected	All in	Marked ventral bend	Mild right bowing	C1 rotated left side ventral	No	No
7	Possibly: defect in dens autolysis or lesion	Not dissected	All in	Mild S-bend	Mild left bowing	C1 angled, not rotated	No	No
8	Yes, especially cranial vertebral SOCs	Not dissected	All in	Straight	Straight	C2 rotated right side ventral	No	No
9	No	Not dissected	All in	Mild ventral bowing	Mild S-bend left, then right	C1 rotated right side ventral	No	No
10 (torticollis)	No	Dissection ok	All in	Dramatic ventral bend centred at C4-C5	Dramatic left bend centred at C4-C5	Dramatic rotation centred at C4-5	No	No (torticollis)
11	Possibly: speckled texture	Not dissected	All in	Straight	Moderate left bowing	Straight	No	No
12	No	Dissection ok	All in	Mild dorsal bowing	Straight	C1 rotated right side ventral	No	No
13	No	Not dissected	All in	Mild S-bend	Straight	C1 rotated right side ventral	No	No
14p	Yes	Dissection ok	All in	Mild dorsal bowing	Mild left bowing	C1 rotated left side ventral	No	No
15	Possibly: tubular defects especially in cranial vertebral SOCs	Not dissected	All in	Mild S-bend	Mild left bowing	Straight	No	No
16	Possibly: slightly speckled texture	Dissection ok	All in	Straight	Straight	C1 rotated left side ventral	No	No
17d	No	Dissection ok	Two acquisitions, all in	Straight	Straight	C1 rotated right side ventral	No	No
18p	No	Not dissected	All in	Mild S-bend	Relatively straight	C1 rotated left side ventral	No	No
19	No	Dissection ok	All in	Very straight	Mild left bowing	Straight	No	No
20	No	Not dissected	All in	Straight	Straight	C1 angled, not rotated	No	No
21	No	Not dissected	All in	Straight	Mild right bowing	Straight	No	No

Case	Decomposition	Dissection	Collimation	Dorso-ventral positioning	Latero-lateral positioning	Rotation	Artefacts	Asymmetry of ventral laminae of C6
22p	No	Dissection ok	All in	Straight	Marked right bowing	C1 rotated right side ventral	Metallic positioning aid at Th1	No
23p	Possibly, slightly speckled texture	No dissect	All in	Straight	Straight	C1 rotated left side ventral	No	No
24	No	Small part of C7 shaved off caudally	All in but small part of C7 missing	Straight	Straight	C1 rotated left side ventral	No	No
25	No	Small part of C7 shaved off caudally	All in but small part of C7 missing	Mild ventral bowing	Straight	C1 rotated left side ventral	No, but fragments at C7 from dissection?	Yes; C6 right ventral lamina large, left lamina small, also: C7 lamina-like structure left side
26	No	Dissection ok	Small part of cranial C1 outside collimation, C7 all in dorsally; caudo-ventral corner outside collimation	Straight	Marked right bowing	C1 rotated left side ventral	No	No
27d	No	C7 missing	Small part of cranial C1 outside collimation C6 all in	Straight	Straight	C1 rotated left side ventral	No	No
28	No	Dissection ok	Small part of cranial C1 outside collimation, C7 all in	Mild ventral bowing	Mild right bowing	Straight	No	No
29	Possibly, slightly speckled texture	Dissection ok	Small part of cranial C1 and caudal half of C7 outside collimation	Straight	Mild S-bend right, then left	C1 rotated left side ventral	Some unknown material next to C6-C7	No
30d	No	Dissection ok	Two acquisitions; all in	Mild S-bend	Mild left bowing	Straight	No	No
31	No	Dissection ok	Small part of cranial C1 outside collimation, C7 all in	Mild ventral bowing	Straight	C1 rotated left side ventral	No artefacts	No
32	No	Dissection ok	Two acquisitions; all in	Straight	Mild left bowing	C1 rotated right side ventral	No	No
33	No	Dissection ok	All in	Mild ventral bowing	Slight right bend	C1 rotated left side ventral	No	No
34	No	Dissection ok	All in	Mild ventral bowing	Mild right bowing	C1 rotated right side ventral	Something linear in scan – catheter?	No
35	No	Dissection ok	Small part of cranial C1 outside collimation, C7 all in	Relatively straight	Straight	C1 rotated left side ventral	No	Yes; SOC absent in left ventral lamina of C6

Abbreviations: SOC: Secondary ossification centre. †p, Premature cases; d, Dysmature cases.
