# Supplementary material for: Closure of the neuro‐central synchondrosis and other physes in foal cervical spines
Source: Equine Vet J. 2024 Apr 9;57(1):217–31. doi: 10.1111/evj.14093 (PMC11616957; doi:10.1111/evj.14093)
Supplement: Supplementary file 4 — Table S3. Vertebral length (cm). Cases are presented in order of increasing mean vertebral length. [file EVJ-57-217-s005.pdf]

Equine Veterinary Journal BEVA

Table S3: Vertebral length (cm). Cases are presented in order of increasing mean vertebral length.

**Case†**	**1**	**2**	**3**	**6**	**8**	**4**	**7**	**5**	**14p**	**16**	**18p**	**20**	**10**	**17d**	**21**	**12**	**19**	**9**	**13**
**Age, days‡**	153	244	244	289	311	271	310	280	0 (320)	0	1	6	335	0 (367)	6	N/r	3	327	N/r
**Breed**	Conn	Arab	Arab	Shet	WB	Ice	WelCo	STB	WelMt	STB	STB	WB	WB	STB	WB	WB	WB	STB	CBT
C1	0.67	1.15	1.79	2.24	2.37	2.81	2.72	2.74	3.74	2.17	3.91	3.95	4.62	3.70	3.30	4.69	3.59	3.98	4.37
C2	0.49	0.85	3.10	4.04	2.88	4.99	4.83	5.13	6.17	7.34	7.57	7.91	7.32	7.66	7.50	7.72	8.27	7.94	7.92
C3	0.47	0.91	1.38	2.44	3.27	3.18	3.26	3.37	3.95	4.90	4.76	4.52	4.76	4.86	4.95	4.77	4.94	4.98	5.04
C4	0.47	0.91	1.26	2.41	3.14	3.00	3.20	3.15	3.81	4.71	4.70	4.74	4.38	4.76	4.85	4.77	4.87	4.89	5.00
C5	0.46	0.86	1.28	2.34	2.99	3.00	3.12	3.16	3.95	4.56	4.52	4.58	4.40	4.79	4.89	4.53	4.80	4.87	4.92
C6	0.46	0.81	1.13	2.20	2.81	2.71	2.94	3.03	3.65	4.41	4.30	4.29	4.19	4.41	4.55	4.29	4.55	4.70	4.59
C7	0.42	0.80	1.02	2.00	2.33	2.34	2.53	2.54	2.99	3.84	3.53	3.86	4.20	3.84	4.05	3.91	4.10	4.17	3.97
Sum	3.44	6.29	10.96	17.68	19.78	22.03	22.59	23.11	28.25	31.93	33.29	33.85	33.86	34.01	34.09	34.67	35.11	35.52	35.81
Mean	0.49	0.90	1.57	2.53	2.83	3.15	3.23	3.30	4.04	4.56	4.76	4.84	4.84	4.86	4.87	4.95	5.02	5.07	5.12

**Case**	**11**	**15**	**23p**	**27d**	**24**	**22p**	**25**	**26**	**28**	**29**	**30d**	**32**	**35**	**31**	**34**	**33**	**Sum**	**Mean**	**Order**
**Age, days†**	N/r	0 (340)	14 (312)	65	20	6 (318)	21	38	93	115	227 (367)	260	438	253	366	316			
**Breed**	WB	WB	WB	STB	Ice	WB	STB	WB	STB	CBT	STB	TB	Fjord	STB	CBT	WB			
C1	4.31	4.61	4.19	4.59	4.82	4.33	4.71	5.43	5.57	5.37	8.28	7.70	9.17	8.17	8.73	9.19	157.66	4.50	6
C2	7.95	8.34	8.18	8.13	8.54	9.00	9.10	10.44	10.75	12.34	13.85	14.10	14.65	15.63	15.16	15.20	290.96	8.31	1
C3	5.05	5.32	5.24	5.26	5.35	5.61	5.70	6.85	6.97	8.40	9.36	9.94	10.72	10.60	11.04	11.19	193.30	5.52	2
C4	5.04	5.00	5.23	5.15	5.35	5.58	5.73	6.91	7.02	8.47	9.17	9.99	10.68	10.73	11.20	10.90	191.13	5.46	3
C5	5.01	4.89	5.09	5.97	5.40	5.55	5.57	6.94	7.05	8.35	9.17	9.98	10.45	10.87	11.00	11.28	189.68	5.42	4
C6	4.47	4.59	4.93	4.68	5.05	5.43	5.25	6.76	6.59	7.85	8.21	9.30	9.30	9.89	10.24	9.80	176.30	5.04	5
C7	4.27	3.93	4.28	N/a	4.33	4.72	4.36	4.61	5.42	N/a	6.86	7.86	7.01	8.66	8.17	8.73	139.63	4.23	7
Sum	36.11	36.68	37.13	32.88	38.84	40.21	40.41	47.92	49.36	50.77	64.89	68.87	71.97	74.53	75.54	76.28			
Mean	5.16	5.24	5.30	5.48	5.55	5.74	5.77	6.85	7.05	8.46	9.27	9.84	10.28	10.65	10.79	10.90			

Abbreviations: Arab: Arabian horse. CBT: Coldblooded trotting horse. Conn: Connemara pony. Fjord: Fjord pony. Ice: Icelandic Horse (pony). N/a: Not applicable (C7 missing from two scans). N/r: Not recorded. Shet: Shetland pony. STB: Standardbred horse. TB: Thoroughbred horse. WB: Warmblood horse. WelCo: Welsh Cob pony. WelMt: Welsh Mountain pony.

†p, Premature cases; d, Dysmature cases. ‡Cases 1-13 were abortions and stillbirths: age is days of gestation. For cases born live, gestation length is given in parenthesis if known.

Blue: 12 Warmblood horses. Green: 10 Standardbred horses. Red: 7 miscellaneous ponies. Yellow: 6 breeds other than Warmblood or Standardbred horses.
