# Supplementary material for: Closure of the neuro‐central synchondrosis and other physes in foal cervical spines
Source: Equine Vet J. 2024 Apr 9;57(1):217–31. doi: 10.1111/evj.14093 (PMC11616957; doi:10.1111/evj.14093)
Supplement: Supplementary file 5 — Table S4A. Radiological maturity—early group. [file EVJ-57-217-s001.pdf]

Table S4A: Radiological maturity – early group.

Cases are presented in the order that the ossification centres formed. The features in the first row were evaluated in terms of size, shape and contours, for example: small ossification centres were ranked as less mature and assigned a higher numerical value than more mature features.

Column 4: Ossification centres were assigned numerical value 4 if they had very small articular processes, 2 if they had small articular processes and 1 if they had clearly formed articular and transverse processes.

Column 5: Ossification centres were assigned value 3 if they were small and generic shape, 2 if they were small cylinders with a ventral crest and 1 if they were large cylinders.

Column 6: Ossification centres were assigned value 2 if they were small and generic shape and 1 if they were conical.

Column 7: Ossification centres were assigned value 3 if they were small and generic shape, 2 if they were small and wedge-shaped and 1 if they were large and wedge-shaped.

Column 8: Ossification centres were assigned value 3 if they were large and round, 2 if they were small and hemispherical with grooves and 1 if they were large and smooth.

Column 9: Ossification centres were assigned value 3 if they were small and generic shape, 2 if they were small and disc-shaped and 1 if they were thin, crescent-shaped and reached the periphery of the vertebral body.

Column 10: Ossification centres were assigned value 4 if they were small, round ossification nuclei, 3 if they were small, conical ossification centres, 2 if they were small hemispherical ossification centres and 1 if they were large hemispherical ossification centres.

Rank	Case	Age†	Primary ossification centre in neural arches C1-C7	Primary ossification centre in vertebral bodies C2-C7	C2 dens ossification centre	Primary ossification centre in the ventral arch of C1	Secondary ossification centre cranially in C3-C7	Secondary ossification centre caudally in C2-C7	Secondary ossification centre cranially in C2
1	1	153	3 very small APs, small TSPs	3 small, generic shape	Absent	Absent	Absent	Absent	Absent
2	2	244	2 small APs, small TSPs	3	Absent	Absent	Absent	Absent	Absent
3	3	244	1 Clear APs and TSPs	2 small cylinder, ventral crest	2 small, generic shape	3 small, generic shape	Absent	Absent	Absent
4	6	289	1	1 large, oblique cylinder	1 conical	2 small, wedge-shaped	3 large, round and uniquely fused	3 small, generic shape	Absent
5	8	311	1	1	1	1 large, wedge-shaped	2 small hemisphere with grooves	3	4 small, round ossification nucleus
6	5	280	1	1	1	1	2	2 small discs, inside periphery	3 small, conical ossification centre
7	7	310	1	1	1	1	2	1 thin crescents to periphery of body	2 small hemisphere
8	4	271	1	1	1	1	1 large, smooth hemisphere	1	1 large hemisphere
9	14p†	0 (320)	1	1	1	1	1	1	1

Abbreviations: APs, Articular processes, TSPs, Transverse processes.
†Age is days of gestation, except case 14p that was born live and died in the first 24 hours, but 12 days premature, equivalent to 320 days of gestation.
