# Supplementary material for: Closure of the neuro‐central synchondrosis and other physes in foal cervical spines
Source: Equine Vet J. 2024 Apr 9;57(1):217–31. doi: 10.1111/evj.14093 (PMC11616957; doi:10.1111/evj.14093)
Supplement: Supplementary file 6 — Table S4B. Middle group. [file EVJ-57-217-s002.pdf]

Table S4B: Middle group.

Cases are presented in order of radiological maturity. Maturity was ranked by counting the number of open dorsal physes and fusion lines visible in 3D-rendered CT images, and by evaluating the size, shape and margins of the ventral laminae of C6 and dorsal spinous process of C7. Features that were interpreted as less mature were assigned higher numerical values than features that were interpreted as more mature.

Column 6: Ventral laminae were assigned numerical value 5 if their shape was only slightly different from C5, value 4 if they were short ridges, value 3 if they were tall ridges, value 2 if they were tall ridges with partial bone lipping (thickening) and value 1 if they were mature shape with bone lipping.

Column 7: Dorsal spinous processes were assigned value 5 if their shape was only slightly different from C6, value 4 if they were protruding, triangular foci, value 3 if they were larger and more square-shaped, value 2 if they were taller and more rectangular-shaped and value 1 if they were mature shape with bone lipping.

Rank	Case[†]	Age[‡]	Number of open dorsal physes visible in 3D-rendered CT images	Number of open fusion lines[§] visible in 3D-rendered CT images	Ventral laminae C6	Dorsal spinous process C7	Sum[¶]
10	17d	0 (367)	6	14	5 shape slightly different from C5	3	28
11	19	3	4	14	5	5 shape slightly different from C6	28
12	9	327	3	13	3 tall ridges	5	24
13	12	Not recorded	5	13	2 tall ridges with partial bone lip[#]	4 protruding focus, triangular	24
14	18p	1	4	11	4 short ridges	3 larger, more square shape	22
15	20	6	5	9	4	4	22
16	11	Not recorded	3	11	5	2 taller, more rectangular	21
17	16	0	2	9	2	5	18
18	10	335	4 (NB torticollis)	6 (NB torticollis)	4	4	18
19	21	6	3	8	5	2	18
20	15	0 (340)	2	9	4	2	17
21	22p	6 (318)	2	6	4	4	16
22	23p	14 (312)	3	5	2	3	13
23	13	Not recorded	2	4	3	3	12
24	24	20	3	7	1 mature shape, bone lipping	1 mature shape, bone lipping	12
25	25	21	3	5	2	2	12

[†]p, Premature cases; d, Dysmature cases. [‡]Cases 9-13 were stillbirths: age is days of gestation. For cases born live, gestation length is given in parenthesis if known. [§]Fusion line, a hypoattenuating line where the lateral portion fused with the medial portion of the primary ossification centre of the neural arch ventral to the transverse foramen (see Fig. 2D). [¶]If several cases had the same sum, the default was to place them in order of increasing age, or decreasing value in most categories if age was not recorded. [#]Bone lip: bone thickening.

Middle group split into an unborn group and a born group, the latter being ordered by increasing age, or increasing size when age was equal

Rank	Case†	Age‡	Number of open dorsal physes visible in 3D-rendered CT images	Number of open fusion lines§ visible in 3D-rendered CT images	Ventral laminae C6	Dorsal spinous process C7	Sum¶
Unborn group							
12	9	327	3	13	3 tall ridges	5	24
13	12	Not recorded	5	13	2 tall ridges with partial bone lip#	4 protruding focus, triangular	24
16	11	Not recorded	3	11	5	2 taller, more rectangular	21
18	10	335	4 (NB torticollis)	6 (NB torticollis)	4	4	18
23	13	Not recorded	2	4	3	3	12
Born group							
20	15	0 (340)	2	9	4	2	17
17	16	0	2	9	2	5	18
10	17d	0 (367)	6	14	5 shape slightly different from C5	3	28
14	18p	1	4	11	4 short ridges	3 larger, more square shape	22
11	19	3	4	14	5	5 shape slightly different from C6	28
15	20	6	5	9	4	4	22
19	21	6	3	8	5	2	18
21	22p	6 (318)	2	6	4	4	16
22	23p	14 (312)	3	5	2	3	13
24	24	20	3	7	1 mature shape, bone lipping	1 mature shape, bone lipping	12
25	25	21	3	5	2	2	12

†p, Premature cases; d, Dysmature cases. ‡Cases 9-13 were stillbirths: age is days of gestation. For cases born live, gestation length is given in parenthesis if known. §Fusion line, a hypoattenuating line where the lateral portion fused with the medial portion of the primary ossification centre of the neural arch ventral to the transverse foramen (see Fig. 2D). ¶If several cases had the same sum, the default was to place them in order of increasing age, or decreasing value in most categories if age was not recorded. #Bone lip: bone thickening.
