# Supplementary material for: Closure of the neuro‐central synchondrosis and other physes in foal cervical spines
Source: Equine Vet J. 2024 Apr 9;57(1):217–31. doi: 10.1111/evj.14093 (PMC11616957; doi:10.1111/evj.14093)
Supplement: Supplementary file 7 — Table S4C. Late group. [file EVJ-57-217-s007.pdf]

Table S4C: Late group.

Cases are presented in order of radiological maturity. Maturity was ranked by evaluating the contour of the condyles of C2 towards C1 and the shape of the secondary ossification centre cranially in C2, as well as the contours of the cranial part of the transverse process of C5 and the caudal physis of C6. Features that were interpreted as less mature were assigned higher numerical value than features that were interpreted as more mature.

Column 4: Condyles were assigned numerical value 5 if they had dimples and tubes generally on C1 and C2 and laterally on the dens, value 4 if they had dimples and tubes centrally on C1 and C2 and regionally on the dens, value 3 if they had dimples and tubes centrally on C1 and regionally on C2, value 2 if they had dimples and tubes regionally on C2 and value 1 if they were sharp and smooth.

Column 5: Ossification centres were assigned value 5 if they were a regular ossification centre, value 4 if they were a square outlined by shallow grooves in the ventral contour of C2, value 3 if part of the margins of the square were granular in appearance, value 2 if all of the margins of the square were granular in appearance and value 1 if only part of the margins of the square were visible and the rest of the margins was merged with C2. Half-values were used when ossification centres were between whole values.

Column 6: The cranial part of the transverse process was assigned value 4 when it was relatively smooth, value 3 when it had shallow dimples and short tubes, value 2 when it had deep dimples and tall tubes, and value 1 when it was smooth.

Column 7: The caudal physis was assigned value 5 when it was smooth with very shallow dimples, value 4 when it had extensive deep dimples centrally and tall tubes, value 3 when it had more limited and shallower central dimples and shorter tubes, value 2 when some of the tubes were coalescing and there were small mineralised bodies and value 1 when there were large coalescing mineralised bodies.

Rank	Case[†]	Age	C2 condyles towards C1	C2 cranial secondary ossification centre	C5 cranial part of transverse process	C6 caudal physis	Sum[‡]
26	28	93 days	5 dimples and tubes generalised on C1 and C2, laterally on dens	4.5	4 relatively smooth	5 smooth, with very shallow dimples	18.5
27	26	38 days	4 dimples and tubes centrally on C1 and C2, regionally on dens	5 ossification centre	4	5	18
28	27d	65 days	4	5	4	5	18
29	29	115 days	4	4 square outlined by shallow grooves in ventral contour C2	3 shallow dimples, short tubes	4 extensive deep central dimples and tall tubes, SOC > POC side	15
30	31	253 days	3 dimples and tubes centrally on C1, regionally on C2, dens smooth	3 part of square margins granular appearance	2 deep dimples, tall tubes	4	12
31	30d	227 days (367 days of gestation)	2 dimples and tubes regionally on C2	3	2	3 limited central shallower dimples and shorter tubes, SOC > POC side	10
32	32	260 days	2	2 all of square margins granular appearance	2	4	10
33	33	316 days	1 sharp and smooth	2	2	4	9
34	34	366 days	1	1.5	2	2 same as 3, but with small coalescing tubes and mineralised bodies	6.5
35	35	438 days	1	1 part of square margins visible, rest merged with C2	1 smooth	1 same as 2, but with large coalescing mineralised bodies	4

[†]p, Premature cases; d, Dysmature cases. [‡]If several cases had the same sum, the default was to place them in order of increasing age or decreasing value in most categories.
