# Supplementary material for: Closure of the neuro‐central synchondrosis and other physes in foal cervical spines
Source: Equine Vet J. 2024 Apr 9;57(1):217–31. doi: 10.1111/evj.14093 (PMC11616957; doi:10.1111/evj.14093)
Supplement: Supplementary file 8 — Table S5. Physeal closure. Cases are presented in order of decreasing mean closure scores. [file EVJ-57-217-s003.pdf]

Equine Veterinary Journal

Table S5: Physeal formation and closure. Cases are presented in order of decreasing mean closure scores.

Case†	1	2	3	8	5	4	6	7	14p	12	16	27d	17d	11	15	19	9	18p	21	25	10	20	13	22p	23p	24	26	28	29	32	31	33	30d	35	34
**Age, days‡**	153	244	244	311	280	271	289	310	0	N/r	0	65	0	N/r	0	3	327	1	6	21	335	6	N/r	6	14	20	38	93	115	260	253	316	227	438	366
**Breed**	Conn	Arab	Arab	WB	STB	Ice	Shet	WelCo	(320) WelMt	WB	STB	STB	(367) STB	WB	(340) WB	WB	STB	STB	WB	STB	WB	WB	CBT	(318) WB	(312) WB	Ice	WB	STB	CBT	TB	STB	WB	(367) STB	Fjord	CBT
**C1 physes**																																			
Dorsal	7	7	7	6	6	6	6	5	5	5	5	5	5	5	5	5	5	5	5	5	5	2.5	5	5	5	5	4	3.5	4	3	3	4	3	1	2
L NCS	9	9	8	7	7	7	7	7	6	6	6	6	6	6	6	6	6	6	6	6	6	5	6	6	6	6	5	5	1	0	0.5	0	0	0	0
R NCS	9	9	8	7	7	7	7	7	6	6	6	6	6	6	6	6	6	6	6	6	6	5	6	6	6	6	5	5	1	0	0.5	0	0	0	0
**C2 physes**																																			
Dorsal	7	7	7	6	5	5	2	3	2	2	2	2	2	2	2	2	2	2	2	1	4	1.5	1	2	1	1	0	1	0	1	1	0	0	0	0
L NCS	6.5	6	6	6	5	5	6	4	3	4	4	3	4	3	3	3	2.5	3	3	2	2	2	3	2	2	2	1	1	1	1	1	1	1	1	0.5
R NCS	6.5	6	6	6	5	5	6	4	4	4	4	3	4	3	3	3	2.5	3	3	2	2	3	3	2	2	2	1	1	1	1	1	1	1	1	0.5
Dens	12	12	11	8	8	6.5	9	8	7.5	6.5	6.5	6	6	6	6	6	6	6	6	6	6	5	5	5	3.5	4	3	3	3	1	1	1	1	1	1
Cranial	9	9	9	8	8	6.5	9	8	7.5	4	4	5	4	5	5	5	4	4	4	4	4	4	4	4	2	2	1	2	1	1.5	1	1	1	1	1
Caudal	9	9	9	8	8	7.5	9	8	6.5	5	5	5	5	5	5	5	5	5	5	5	4	4.5	4.5	4	3	2	4	2.5	4	3	3	3	3	3	3
**C3 physes**																																			
Dorsal	7	7	7	6	3	2	2	2	2	2	2	2	2	1	1	2	1	1	1	0	0	2	0.5	0	1	0	0	0	0	0	0	0	0	0	0
L NCS	6.5	6	6	6	5	5	6	4	2	3	4	3	2	3	2.5	2	2	2	2	2	2	2	2.5	2	1.5	2	1	1	1	1	1	1	1	1	0
R NCS	6.5	6	6	6	5	5	6	4	2	3	4	3	2	3	2.5	2	2	2	2	2	2	2	2.5	2	1.5	2	1	1	1	1	1	1	1	1	0
Cranial	9	9	9	7	7	7	2	6	6	5	5	4	5	5	5	5	5	4	4	4	3	4	4	4	2	2	2	2	2	2	2	2	2	2	2
Caudal	9	9	9	8	8	7.5	8	7.5	6.5	5	5	5	5	5	5	5	5	5	5	5	3	4	4	4	3	2	3	3	3	3	3	2.5	3	3	3
**C4 physes**																																			
Dorsal	7	7	7	6	3	2	1	2	3	2	2	2	2	0	1	1	1	2	0	1	2	2	0	1	1	0	0	0	0	0	0	0	0	0	0
L NCS	6.5	6	6	6	5	5	6	3	2	4	3	3	2	3	2	2	2	3	2	2	2	2	2	2	2	2	1	1	1	1	1	1	1	1	0
R NCS	6.5	6	6	6	5	5	6	3	2	4	4	3	2	3	2	2	2	3	2	2	2	2	2	2	2	2	1	1	1	1	1	1	1	1	0
Cranial	9	9	9	7	7	7	2	6	6	5	5	4	5	5	5	5	5	4	4	4	3	4	4	4	2	2	3	2	3	2	2	2	2	2	2
Caudal	9	9	9	8	8	7.5	8	7.5	6.5	5	5	5	5	5	5	5	5	5	5	5	3	4	4	4	3	2	4	2.5	4	3	2.5	2.5	3	3	3
**C5 physes**																																			
Dorsal	7	7	7	6	5	2.5	0	2	3	2	2	2	2	0	1	1	2	2	0	1	2	2	0	0	1	0	0	0	0	0	0	0	0	0	0
L NCS	6.5	6	6	6	5	5	6	4	2	4	3	3	2	3	2	2	2	2	2	2	2	2	2	2	2	2	1	1	1	1	1	1	1	1	0
R NCS	6.5	6	6	6	5	5	6	4	2	3	3	3	2	3	2	2	2	2	2	2	2	2	2	2	2	2	1	1	1	1	1	1	1	1	0
Cranial	9	9	9	7	7	7	2	6	6	5	5	4	5	5	5	5	5	4	4	4	4	4	4	4	2	2	2	2	2	2	2	2	2	2	2
Caudal	9	9	9	8	8	7.5	8	7.5	6.5	5	5	5	5	5	5	5	5	5	5	5	4	4	4	4	2.5	2	3	2.5	3	3	2.5	2.5	3	2.5	3
**C6 physes**																																			
Dorsal	7	7	7	6	5	2.5	0	2	3	2	2	2	2	0	0	1	0	1	1	0	2	2	0	0	0	0	0	0	0	0	0	0	0	0	0
L NCS	6.5	6	6	6	5	5	6	3	2	4	3	3	2	2.5	2	2	2	2	2	2	2	2	2	2	1.5	1	1	1	1	1	1	1	1	1	0
R NCS	6.5	6	6	6	5	5	6	3	2	4	3	3	2	2.5	2	2	2	2	2	2	2	2	2	2	1.5	1	1	1	1	1	1	1	1	1	0
Cranial	9	9	9	7	7	7	2	6	6	4	5	4	5	5	5	5	5	4	4	4	4	4	4	4	2	2	2	1.5	2	2	2	2	2	1.5	2
Caudal	9	9	9	8	8	7.5	8	7.5	6.5	5	5	5	5	5	5	5	5	5	5	5	4	4	4	4	2.5	2	2.5	2	3	3	2.5	2.5	3	2	3
**C7 physes**																																			
Dorsal	7	7	7	6	3	2	0	1	1	1	1	N/a	1	0	0	0	1	1	1	0	0	0	0	0	0.5	0	0	0	N/a	0	0	0	0	0	0
L NCS	6.5	6	6	6	5	4	6	4	2	4	3	N/a	2	2	2.5	2	2	2.5	2	2	2	2	2	2	1.5	2	1	1	N/a	1	1	1	0	1	0
R NCS	6.5	6	6	6	5	5	6	4	2	3	3	N/a	2	2.5	2.5	2	2	2.5	2	2	2	2	2	2	1.5	2	1	1	N/a	1	1	1	0	1	0
Cranial	9	9	9	7	7	7	5	6	6	4	5	N/a	5	5	5	5	5	4	4	4	4	4	4	4	1	2	2	1	N/a	2	2	2	2	1	2
Caudal	9	9	9	8	8	7.5	8	7.5	6.5	5	5	N/a	5	5	5	5	5	4	5	5	4	4	4	4	1.5	2	2	2	N/a	3	2.5	2.5	3	2	3
**Sum**	265	259	256	227	203	188	177	166.5	142	135.5	134.5	109	121	119.5	116	116	114	114	108	104	101	100.5	99	97	72.5	68	59.5	54.5	46	46.5	45	43.5	43	39	33
**Mean**	7.79	7.62	7.53	6.68	5.97	5.53	5.21	4.90	4.18	3.99	3.96	3.76	3.56	3.51	3.41	3.41	3.35	3.35	3.18	3.06	2.97	2.96	2.91	2.85	2.13	2	1.75	1.60	1.59	1.37	1.32	1.28	1.26	1.15	0.97

Abbreviations: Arab: Arabian horse. CBT: Coldblooded trotting horse. Conn: Connemara pony. Fjord: Fjord pony. Ice: Icelandic Horse (pony). L, Left. N/a: Not applicable (C7 missing from two scans). NCS, Neuro-central synchondrosis. N/r: Not recorded. R, Right. Shet: Shetland pony. STB: Standardbred horse. TB: Thoroughbred horse. WB: Warmblood horse. WelCo: Welsh Cob pony. WelMt: Welsh Mountain pony.

†p, Premature cases; d, Dysmature cases. ‡Age: Cases 1-13 were abortions and stillbirths: age is days of gestation. For cases born live, gestation length is given in parenthesis if known.

Red, scores 12-6.5: FORMING. Orange, scores 6-3.5: OPEN. Yellow, scores 3-1.5: CLOSING. Green, scores 1-0: CLOSED.
